# Fluorescent Nanozeolite Receptors for the Highly Selective and Sensitive Detection of Neurotransmitters in Water and Biofluids

**DOI:** 10.1002/adma.202104614

**Published:** 2021-09-27

**Authors:** Laura M. Grimm, Stephan Sinn, Marjan Krstić, Elisa D'Este, Ivo Sonntag, Eko Adi Prasetyanto, Thomas Kuner, Wolfgang Wenzel, Luisa De Cola, Frank Biedermann

**Affiliations:** ^1^ Institute of Nanotechnology Karlsruhe Institute of Technology (KIT) Hermann‐von‐Helmholtz Platz 1 76344 Eggenstein‐Leopoldshafen Germany; ^2^ Max‐Planck‐Institute for Medical Research Jahnstraße 29 69120 Heidelberg Germany; ^3^ Department of Functional Neuroanatomy Institute for Anatomy and Cell Biology Heidelberg University 69120 Heidelberg Germany; ^4^ Institut de Science et d'Ingénierie Supramoléculaires University of Strasbourg 8 rue Gaspard Monge Strasbourg 67000 France; ^5^ Department of Pharmacy, School of Medicine and Health Sciences Atma Jaya Catholic University of Indonesia Jl. Pluit Raya no 2 Jakarta 14440 Indonesia; ^6^ HEiKA ‐ Heidelberg Karlsruhe Strategic Partnership Heidelberg University Karlsruhe Institute of Technology (KIT) 76134 Karlsruhe Germany; ^7^ Istituto di Ricerche Farmacologiche Mario Negri IRCCS Via Mario Negri, 2 Milan 20156 Italy

**Keywords:** artificial receptors, biofluids, diagnostic assays, nanoparticles, neurotransmitter, sensors, zeolites

## Abstract

The design and preparation of synthetic binders (SBs) applicable for small biomolecule sensing in aqueous media remains very challenging. SBs designed by the lock‐and‐key principle can be selective for their target analyte but usually show an insufficient binding strength in water. In contrast, SBs based on symmetric macrocycles with a hydrophobic cavity can display high binding affinities but generally suffer from indiscriminate binding of many analytes. Herein, a completely new and modular receptor design strategy based on microporous hybrid materials is presented yielding zeolite‐based artificial receptors (ZARs) which reversibly bind the neurotransmitters serotonin and dopamine with unprecedented affinity and selectivity even in saline biofluids. ZARs are thought to uniquely exploit both the non‐classical hydrophobic effect and direct non‐covalent recognition motifs, which is supported by in‐depth photophysical, and calorimetric experiments combined with full atomistic modeling. ZARs are thermally and chemically robust and can be readily prepared at gram scales. Their applicability for the label‐free monitoring of important enzymatic reactions, for (two‐photon) fluorescence imaging, and for high‐throughput diagnostics in biofluids is demonstrated. This study showcases that artificial receptor based on microporous hybrid materials can overcome standing limitations of synthetic chemosensors, paving the way towards personalized diagnostics and metabolomics.

## Graphical abstract

Fluorescent artificial receptors that bind neurotransmitters with unprecedented affinity and selectivity in biofluids are introduced. They are based on nanozeolite hybrid materials and can be used for emission‐ and absorbance‐based detection of serotonin and dopamine in urine at physiological relevant concentration, for (two‐photon) imaging, and for label‐free enzymatic reaction monitoring in real time.

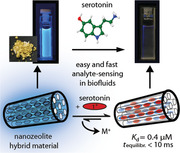

## 1 Introduction

**Figure 1:**
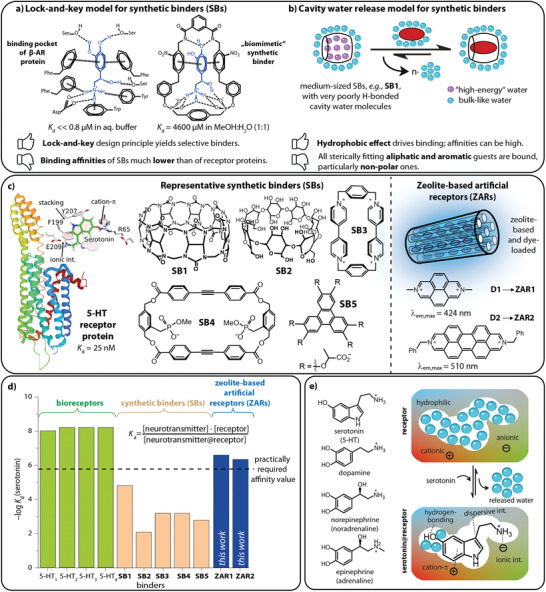
Design strategies for receptors and SBs for neurotransmitters. a) Biomimetic design of SBs often follows the lock‐and‐key principle.^[^ ^18^ ^]^ The example shows norepinephrine binding to left, a β‐adrenergic receptor (β‐AR), and right, a designed SB. b) The cavity water release model provides an alternative design strategy for SBs.^[^ ^15^ ^]^ c) Schematic representation of a bioreceptor of the 5‐HT family and its binding pocket for serotonin (left). For comparison, structures of prominent SBs (middle) for serotonin, and the herein introduced ZARs (right) are shown. ZARs are composed of zeolite L nanoparticles^[^ ^35^, ^36^ ^]^ and encapsulated fluorescent reporter dyes, **D1** (→**ZAR1**) or **D2** (→**ZAR2**). d) Comparison of the binding affinity for serotonin (depicted as log (1/*K* *_d_*) of 5‐HT bioreceptors (green; Table S3, Supporting Information), a selection of the strongest binding SBs known so far (orange, **SB1**‐**SB5**; Table S7, Supporting Information), and the herein introduced ZARs (blue). None of the SBs reaches the practical affinity requirements, not even in desalinated water or aqueous‐organic solvent mixtures. e) Chemical structures of neurotransmitters and schematic depiction of the herein pursued receptor design strategy that combines direct non‐covalent binding motifs (lock‐and‐key elements) and high‐energy cavity water release (non‐classical hydrophobic effect).

Artificial receptors and nanosensors are envisioned to open exciting new possibilities for home‐use and point‐of‐care diagnostics as they can be chemically/thermally more robust, inexpensive, and faster responding than complementary biosensors.^[^ ^1^, ^2^, ^3^, ^4^, ^5^, ^6^, ^7^, ^8^, ^9^, ^10^ ^]^ Inspiring examples are the molecular recognition‐based glucose sensors developed separately by Senseonics and GlySure Ltd^[^ ^11^, ^12^ ^]^ and cation‐selective chemosensors used in a supramolecular sensor cassette by OPTI Medical Inc for Na^+^, K^+^, and Ca^2+^ sensing in blood.^[^ ^13^, ^14^ ^]^ Nevertheless, the selective and sensitive molecular recognition of small hydrophilic molecules in water remains extremely challenging (**Figure** **1**).^[^ ^15^, ^16^, ^17^ ^]^ For instance, synthetic binders (SBs) designed to recognize the neurotransmitter dopamine through direct non‐covalent binding motifs, for example, salt bridges and stacking interactions (Figure 1a), are relatively selective for their target molecules but are limited by impractically low binding affinities in water.^[^ ^18^ ^]^ In recent years, fundamental and circumstantial evidence was collected showing that the (non‐classical) hydrophobic effect is an important driving force for host‐guest complex formation in water (Figure 1b).^[^ ^16^, ^17^, ^19^, ^20^ ^]^ While the underlying physical details are still under debate,^[^ ^20^, ^21^, ^22^ ^]^ it is empirically clear that SBs with a persistent, shielded, and hydrophobic binding cavity provide the highest affinities.^[^ ^15^, ^17^, ^20^ ^]^ However, even cucurbit[*n*]urils that bind a wide range of hydrophobic compounds with record‐high affinities^[^ ^23^, ^24^ ^]^ fall short with respect to the required *K*_d_ values for serotonin or dopamine recognition at physiologically relevant concentrations (Figure 1b–d). Moreover, such symmetric macrocycle‐based systems are generally unselective binders and complex many metabolites and drugs, both aliphatic and aromatic ones, and are often perturbed by the salts present in biofluids. Nanosensors, for example, based on surface‐functionalized gold nanoparticles or carbon dots that can take advantage of multivalency effects,^[^ ^25^ ^]^ have been reported as alternative designs for the detection of serotonin and dopamine. However, to our knowledge, they do not fulfill the affinity and selectivity requirements for diagnostic applications in biofluids.^[^ ^26^, ^27^, ^28^ ^]^

We assess that all contemporary SBs for serotonin and dopamine (Figure 1, Figure S3 and Table S7, Supporting Information) that were rationally designed performed very poorly,^[^ ^1^, ^29^, ^30^, ^31^, ^32^, ^33^, ^34^ ^]^ because *i*) their affinity for the target molecules is too low, particularly in aqueous media and in the presence of salts; *ii*) their binding selectivity is insufficient, for example, they cannot distinguish neurotransmitters from amino acids; and/or *iii*) they lack in a signal transduction mode and therefore are no receptors. Clearly, the currently pursued design principles for SBs need to be complemented with novel strategies.

Zeolites, which are crystalline materials with highly defined pore dimensions (Table S1, Supporting Information),^[^ ^37^, ^38^ ^]^ appear particularly promising because they are available on a tons‐scale and have previously been used for catalytic and biomedical applications.^[^ ^35^, ^39^, ^40^, ^41^, ^42^, ^43^ ^]^ Their framework is composed of corner‐sharing [SiO_4_]‐ and [AlO_4_]‐tetrahedra and possesses an overall negative framework charge that is neutralized by exchangeable cations, for example, Na^+^ and K^+^. Thus, zeolites bind positively charged guests, for example, biogenic amines like cadaverine. However, they display an insufficient affinity for the positively charged neurotransmitter serotonin in water and do not provide a direct optical response.

Here, we investigate porous inorganic materials to construct artificial receptors since their cavities are shielded from bulk solvent contact and are persistent in shape in combination with dicationic dyes. Cations bound inside the negative inorganic materials can be replaced by positively charged dye molecules introducing an intramolecular signaling unit. As a result of the interplay of hydrophobic effect, ionic interactions, cation‐π‐interactions, and hydrogen bonding, very high binding affinities and selectivity for hydrophilic neurotransmitters such as serotonin can be reached (Figure 1e).

## 2 Results and Discussion

### 2.1 Design and Preparation of Zeolite‐Based Artificial Receptors (ZARs)

We hypothesized that inorganic porous materials can provide a promising scaffold for the strong and selective capture of both charged, small, hydrophilic metabolites and complementary sensitive, specific, and selective fluorescent probes. Indeed, our porous chemosensors are biocompatible, possess a controlled and permanent pore size and a tunable framework charge and functionality, thereby excluding the interference of any biomacromolecules (size‐selection‐principle), and non‐complementary charged metabolites (charge‐selection‐principle). Furthermore, the persistent cavity shape and complete shielding of bulk solvent contact allows for efficiently exploiting the hydrophobic effect as driving force for binding.

**Figure 2:**
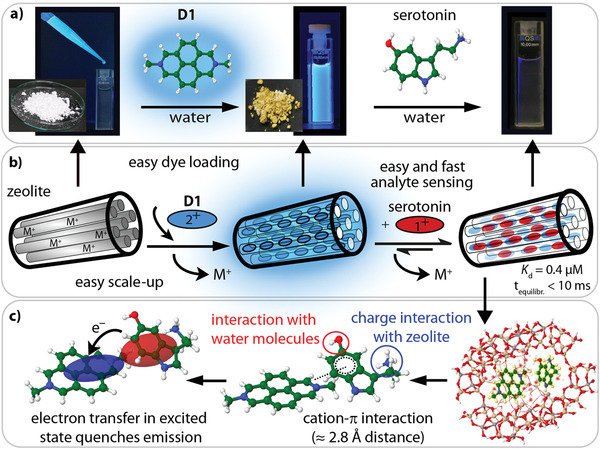
Preparation of and sensing with ZARs. ZARs can be easily prepared on a gram scale through the impregnation of dicationic reporter dyes inside zeolite L nanoparticles. a) Photographs of the different steps of the ZAR synthesis and serotonin detection, monitored by emission quenching. The procedure is schematized in (b). c) The binding geometry and the emission quenching mechanism between dye and neurotransmitter in the zeolite channels are obtained by full atomistic DFT calculations, where cavity water molecules are explicitly considered (Figure S32–S35, Supporting Information).

Specifically, Linde‐type zeolite L nanoparticles (50–200 nm particle size, channel entrance diameter 7.6 Å) were used as water‐dispersible receptor scaffolds.^[^ ^36^ ^]^ To both render the channels as a perfect environment for the binding of aromatic amine neurotransmitters and to provide an optical signal transduction mechanism, the nanoporous zeolites were loaded with the dicationic fluorescent dyes **D1** and **D2** (Figure 1c). The fluorescent artificial receptors (ZARs) produced in this way realize a design space that combines molecular recognition through both the zeolite pore and the dicationic dye with a signal response to neurotransmitter binding (**Figure** **2**). The dyes **D1** and **D2** were selected because they *i*) fit well into the channels, *ii*) are strongly bound to the negatively charged zeolite channel walls because of cation exchange, and *iii*) tend to be monodispersed inside the channels. Moreover, they *iv*) engage in direct non‐covalent interactions with the neurotransmitters, ensuring the binding strength and selectivity of ZARs, and *v*) possess excellent electron‐accepting properties. Thus, their electronic coupling with neurotransmitters (electron donors) leads to photoinduced electron transfer processes that can be sensitively monitored by fluorescence and absorbance spectroscopy,^[^ ^44^ ^]^ and that are specific for each dye‐neurotransmitter pair.

**Figure 3:**
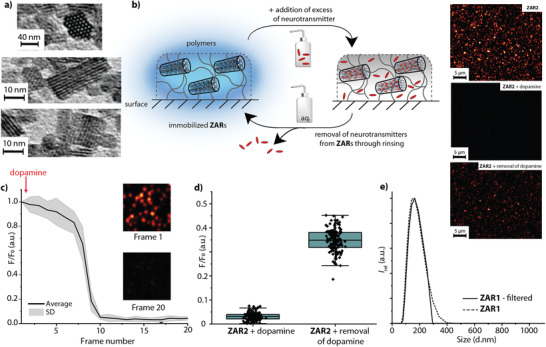
Characterization of ZARs. a) Characterization of ZAR particles by TEM, visualizing the pore openings and the 1D zeolite L channels. b) Schematized representation of the reversible neurotransmitter binding to immobilized ZARs and confirmation by confocal microscopy (images shown with the same brightness). c) Emission quenching of chemosensor **ZAR2** upon the addition of dopamine (*c* = 50 µM) is very fast also for surface‐bound ZARs (time spacing of ≈0.9 s between each image frame, total movie length for 20 frames ≈18 sec). d) Quantification and statistics for the relative emission intensity of surface bound **ZAR2** particles in the presence of dopamine and after (partial) removal of dopamine through rinsing. In contrast to solution experiments where ZAR particles are monodispersed, also clusters of ZAR particles are observed on the surface. e) The size range of the individual ZAR nanoparticles is determined by DLS as approx. 50–200 nm. Filtration is done with a 0.45 µM syringe filter.

Having prepared ZARs by ion exchange in solution and after full characterization by TEM, DLS (**Figure** **3**), zeta potential measurements (Table S2, Supporting Information), and elemental analysis, we investigated their interaction with neurotransmitters further. Due to the small size and lack of aggregation of the particles in water, the solution remained clear for several days and did not scatter visible light in the absorbance spectra. The aqueous dispersions show a bright emission in the visible region upon excitation of the dye. Neurotransmitter uptake and release experiments monitored by confocal and two‐photon microscopy with surface‐immobilized ZARs confirmed the reversible nature of the neurotransmitter binding by repeated additions of neurotransmitter (Figure 3 and Figure S11, Supporting Information).

### 2.2 Binding Parameters and Proposed Binding Mechanism of ZARs

**Figure 4:**
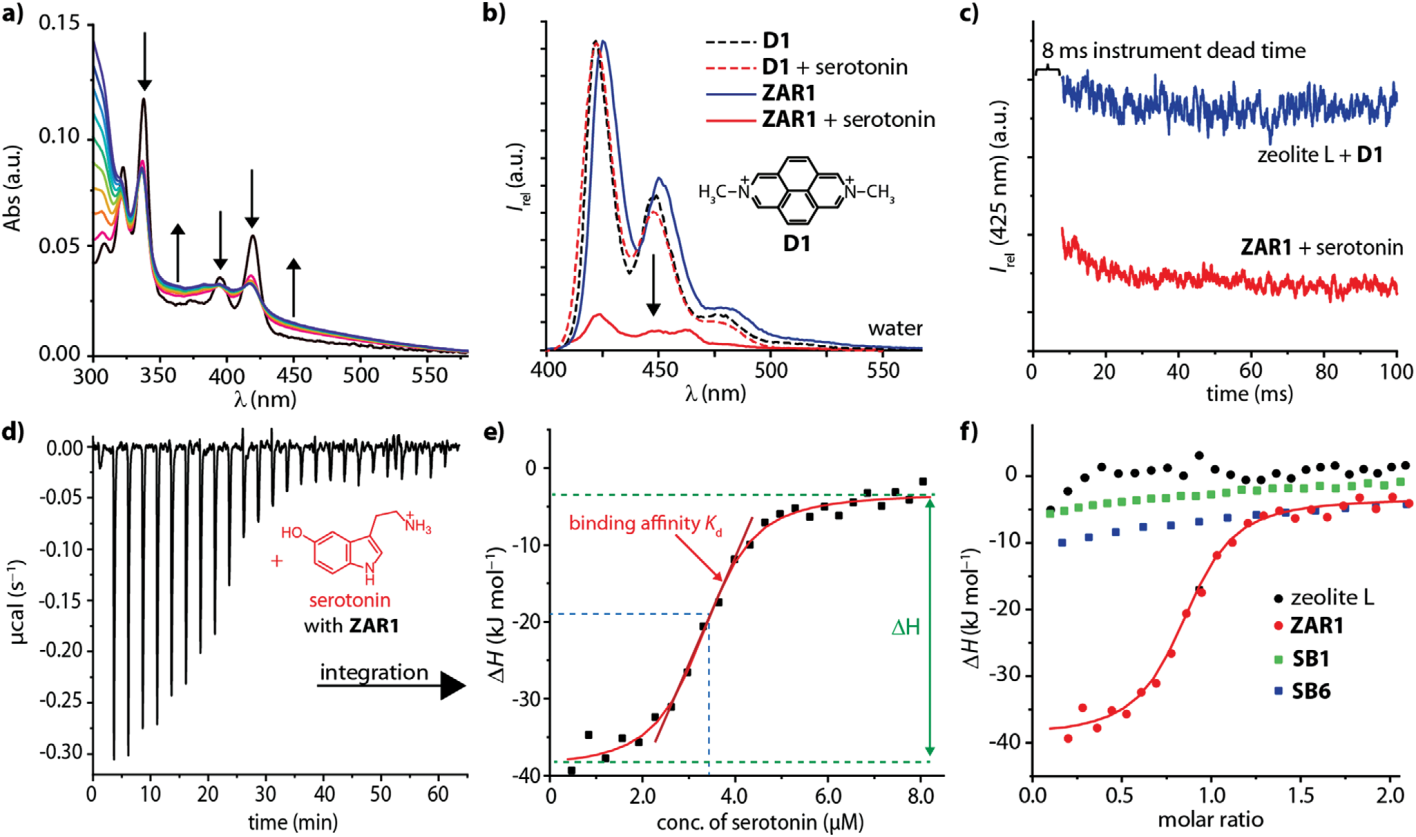
Binding features of ZARs. a) Absorbance‐based titration monitoring of the addition of serotonin leading to a strongly altered absorbance spectrum of **ZAR1**. b) Normalized emission spectra of D1 and its corresponding chemosensor **ZAR1** in the absence and presence of serotonin (λ_ex_ = 371 nm) in water. Serotonin quenches the emission of **ZAR1**, but it does not interact with D1 in aqueous media. c) Kinetic traces for the rapid mixing of serotonin with **ZAR1** (red) in MilliQ water (λ_ex_ = 371 nm) in stopped‐flow experiments. The binding kinetics are found to be very fast (signal saturation < 10 milliseconds). As reference, the kinetic trace for the rapid mixing of D1 with zeolite L is given (blue). Please note that the manufacturer specifies the instrument‐specific dead time to be 8 milliseconds. d,e) ITC thermogram for the titration of serotonin to **ZAR1** nanoparticles displaying a strong exothermic binding signature with a clear 1:1 binding stoichiometry of serotonin to reporter dye D1. f) Comparison of the integrated ITC thermograms of serotonin binding to zeolite L (black), **ZAR1** (red), **SB1** (green), and **SB6** (blue).

Upon titrating the ZAR dispersion with serotonin, a strong emission quenching and a concomitant growing of a charge transfer band (≈430–550 nm) in the absorbance spectra were observed (**Figure** **4**a and Figure S8–S10, Supporting Information), indicative of a photoinduced electron transfer process. This reaction is promoted by a cation‐π‐type dye‐neurotransmitter interaction inside the zeolite L channels, as evidenced by full atomistic density functional theory (DFT) simulations (Figure 2 and Figure S32–S35, Supporting Information). Through time‐dependent DFT (TD‐DFT) simulations and subsequent electron density difference analysis, the physical origin of the emerging charge transfer bands in the absorbance spectra was confirmed to be an excited state electron transfer process that requires neurotransmitters with an electron‐rich aromatic moiety such as serotonin. The binding model is further evidenced by the 1:1 binding stoichiometry found in all spectroscopic and calorimetric titration experiments. Binding affinities were determined by fluorescence and absorbance titration experiments by using Equation (1), assuming that only the ZARs and the ZAR∙neurotransmitter (NT) complexes are emissive at the chosen emission wavelength:

(1) $$\frac{F_{NT}}{F_{0}}=1+\frac{\Delta F\left[\left(c_{NT}+c_{ZAR}+K_{d}\right)-\sqrt{{\left(c_{NT}+c_{ZAR}+K_{d}\right)}^{2}-4\cdot c_{NT}\cdot c_{ZAR}}\right]}{2\cdot c_{ZAR}}$$

where *F*_NT_ is the intensity at a given neurotransmitter (NT) concentration and *F_0_* is the emission intensity before neurotransmitter addition. Δ*F* is a measure of the relative emission increase or decrease caused by the neurotransmitter. For fully non‐emissive ZAR∙NT complexes, that is, when the neurotransmitter NT is an efficient quencher, Δ*F* reaches −1. The quantity *c*_NT_ denotes the concentration of the neurotransmitter NT and *c*_ZAR_ denotes the concentration of the “binding sites” in the ZAR. The values *K*_d_ and Δ*F* result from the non‐least square fit given the input *F*_NT_, *F*_0_, *c*_NT_, and *c*_ZAR_.

High affinities, not as high as that of natural neuroreceptor proteins but much larger than that of any SB, were found for the interactions of ZARs with serotonin and dopamine in deionized water, in saline buffers, and in biofluids (Figure 1d, Figure S3, Figure S12–S18, and Table S5, Supporting Information).

Ratiometric sensing can be implemented by co‐inclusion of a spectator dye whose emission is not affected by the presence of neurotransmitters (Figure S6–S7, Supporting Information). Owing to the high affinities, and to the high sensitivity of the emission signal response, even low neurotransmitter concentrations, for example, 50 nM serotonin, can be detected by ZARs (Figure S7, Supporting Information). Furthermore, the binding kinetics were found to be very fast (signal saturation <10 milliseconds measured by rapid mixing in a stopped‐flow experiment, Figure 4c), which is similar to the response time of natural neuroreceptors and their mutants,^[^ ^45^, ^46^, ^47^ ^]^ but much faster than the equilibration time (min to h) of serotonin‐binding antibodies.^[^ ^48^ ^]^

Further insights into the binding mechanism were obtained by isothermal titration calorimetry (ITC) which measures the heat that is released upon complex formation and offers therefore an independent determination method compared to the photophysical investigations (Figure 4d–f and Figure S23–S26, Supporting Information). The fitting was conducted according to the Wiseman isotherm:^[^ ^49^ ^]^

(2) $$\begin{array}{l} \frac{dQ}{d{\left[NT\right]}_{t}}=\Delta HV_{0}\left[\frac{1}{2}+\frac{1-X_{R}-r}{2\sqrt{{\left(1+X_{R}+r\right)}^{2}-4X_{R}}}\right]l\\ \text{with}\frac{1}{r}=c=K_{a}{\left[M\right]}_{t}=\frac{{\left[M\right]}_{t}}{K_{d}} \end{array}$$

where (*dQ/d[NT*]_t_) refers to the moles of neurotransmitter added per injection, *Χ*_R_ to the absolute ratio of neurotransmitter to ZAR “binding sites” concentration, *c* is the Wiseman parameter, and *V*_0_ is the effective volume of the calorimeter cell. Pleasingly, the binding affinity for serotonin and **ZAR1** by ITC, *K*_d_ = 0.3 ± 0.1 µM, is in excellent agreement with the fluorescence‐based affinity value, *K*_d_ = 0.4 ± 0.1 µM, indicating that serotonin binding next to the reporter dye is the predominant interaction mode and that serotonin does not or only weakly bind to other, non‐dye filled zeolite cavities. Moreover, serotonin binding to **ZAR1** is strongly enthalpically favored (exothermic; Δ*H* = −39.0 ± 2 kJ mol^−1^; Δ*G* = −38.0 ± 2 kJ mol^−1^, and −TΔ*S* = −1.0 ± 2 kJ mol^−1^), whereas the thermodynamic signature of serotonin and the parent nanozeolite L material is that of a weak binding process (Figure 4f). In contrast, the interaction of cadaverine with **ZAR1** or parent zeolite L shows endothermic injection peaks that do not display peak saturation with increasing analyte concentration (Figure S15, Supporting Information). This behavior is indicative for cadaverine binding to non‐dye filled cavities in the microporous framework and corroborates the finding that cadaverine does not adversely affect the fluorescence‐based detection of serotonin by **ZAR1**. Furthermore, the SBs **SB1** and **SB6** only reached binding affinities of *K*_d_ ≥ 14 µM (**SB1**) and *K*_d_ ≥ 200 µM (**SB6**) with Δ*H* ≥ −15.0 ± 2 kJ mol^−1^ for serotonin as analyte (Figure 4f), which is charged and hydrophilic, and thus energetically costly to desolvate. For comparison, the binding of hydrophobic analytes by cavity water release‐type SBs is much more exothermic, for example, Δ*H* = −52.2 ± 2 kJ mol^−1^ for **SB1** and nortestosterone,^[^ ^50^ ^]^ and Δ*H* = −56.5 ± 1 kJ mol^−1^ for **SB6** and indole.^[^ ^51^ ^]^ It is also worth mentioning that serotonin binding by **ZAR1** is considerably weaker in ethanol than in water as a solvent, further indicating the importance of hydrophobic contributions to the ZAR binding mechanism (Figure S30, Supporting Information). Taking all these findings together, we propose that the combination of lock‐and‐key elements, for example, electrostatic attraction between the neurotransmitters and the zeolitic framework, a cation‐π interaction between the dye and the neurotransmitter, and the release of residual cavity water molecules from ZARs are important contributors to the experimentally observed strongly favorable binding enthalpies and binding free energies for neurotransmitter binding by ZARs. Figure S1, Supporting Information, provides a schematic representation which depicts cavity water molecules adjacent to the reporter dye that are due to geometric restrains likely more energetically “frustrated” than other zeolitic cavity water molecules. Thus, the release into the bulk upon serotonin binding causes the strongly exothermic binding signature. Interestingly, strongly entropically favored (endothermic) binding characteristics were reported for the interaction of natural receptor protein 5‐HT_3_ with serotonin,^[^ ^52^ ^]^ completely opposite to the thermodynamic signature of serotonin complexation by **ZAR1**. Hence, this study reveals that it is possible to arrive at nearly protein‐like affinities for small molecules through an artificial, non‐biomimetic design principle and binding mechanism.

### 2.3 ZAR‐Based Neurotransmitter Distinction

**Figure 5:**
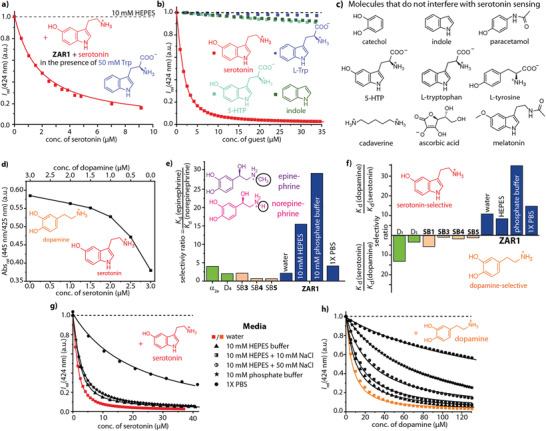
Binding selectivity of ZARs. a) Binding curves for the titration of **ZAR1** with serotonin in 10 mM HEPES buffer, pH 7.3, in the presence of a large excess of L‐Trp (*c* = 50 mM) as potential interferent. b) Binding titration experiments monitored by fluorescence spectroscopy at λ_em_ = 424 nm for **ZAR1** with serotonin and its weakly binding precursors L‐Trp and 5‐HTP additionally to their structural base indole (λ_ex_ = 371 nm). c) Chemical structures of analytes that do not interfere with the serotonin/dopamine sensing. d) Ratiometric absorbance signals can be used for the differentiation of mixtures of dopamine and serotonin by **ZAR1** through exploiting the emerging characteristic charge transfer bands at 445 nm (referenced to the isosbestic point at 425 nm). e,f) Comparison of the selectivity ratios for the binding of e) epinephrine versus norepinephrine and f) serotonin versus dopamine to bioreceptors (green; Tables S3 and S6, Supporting Information), known SBs (orange; Tables S6 and S7, Supporting Information), and **ZAR1** (blue; Table S5, Supporting Information) in water and buffered aqueous media. g,h) Binding titrationexperiments monitored by fluorescence spectroscopy at λ_em_ = 424 nm for FAR1 with g) serotonin and h) dopamine as function of the salinity ofthe aqueous medium.

Applicable artificial receptors must target analytes specifically, and therefore, ZARs were exposed to potential interferents such as amino acids, ascorbic acid, carbohydrates, and biogenic amines, all of which did not cause a signal response. Indeed, the selectivity of ZARs is so high that for instance 5 µM of serotonin can be clearly detected even in the presence of 50 000 µM L‐tryptophan (**Figure** **5**). Likewise, ZARs are not affected by other amino acids, or non‐charged organic compounds such as carbohydrates, the hormone melatonin, the drug paracetamol, or the toxin indole. Even the biogenic amine cadaverine does not interfere at a physiological concentration range with the sensing of serotonin, in spite of its simultaneous binding to ZARs (Figure S13–S14, Supporting Information). Likely, cadaverine occupies vacant, non‐dye‐filled channels. In contrast, known SBs are unselective or even preferentially bind amino acids or biogenic amines, preventing their practical use for neurotransmitter detection. Equally important, the detection of low micromolar levels of serotonin was found to be possible in the presence of different salt concentrations. To simulate typical salt concentrations in urine 0–300 mM NaCl were added to the investigated urine samples (Figure S15, Supporting Information). Thus, undesired matrix‐to‐matrix effects for the ZAR‐based serotonin detection in biofluids are expected to be low. Additionally, the ZAR‐based sensing protocol is tolerant towards redox‐active compounds such as ascorbic acid (Figure S16, Supporting Information), which is known to disturb electrochemical detection methods for dopamine.^[^ ^26^, ^53^ ^]^

For diagnostic applications it is often sufficient to indicate an abnormal *total* neurotransmitter level,^[^ ^54^ ^]^ nevertheless, we wondered if neurotransmitters can be distinguished from each other with our ZARs. Indeed, we found that the dye‐neurotransmitter interaction leads to a very specific electronic coupling, which manifests itself in different levels of emission quenching and the rise of characteristic charge transfer absorbance bands (Figure 4a). These spectroscopic fingerprints can be utilized to distinguish, for example, serotonin from dopamine, and to deconvolute their concentration ratio in mixtures (Figure 5d). Hence, besides the size and charge of the nanoporous scaffold, the dyes play an important role in determining the signal efficacy of ZARs through controlling the electron transfer process. ZARs thereby mimic the functionality of natural receptor proteins that discriminate between ligands by *i)* narrowing down the number of potential binding ligands through molecular recognition (lock‐and‐key principle) and *ii)* providing different efficacies amongst the bound ligands (agonism, neutral agonism, and inverse agonism) (Figure 1 and Figure S3, Supporting Information).^[^ ^55^ ^]^ Interestingly, the neurotransmitters norepinephrine and epinephrine, that differ only by one methyl group, can be readily distinguished in ZAR‐based assays (selectivity ratios up to 28 in saline buffers; Figure 5e and Figure S12, Supporting Information). The binding selectivity of ZARs largely exceeds that of the natural α_2A_‐adrenergic receptor and dopamine D_4_‐receptor proteins (selectivity ratio <5, Table S4 and S6, Supporting Information) for these homologous catecholamines.^[^ ^56^, ^57^ ^]^ The selectivity of SBs is expectedly even lower (selectivity ratio <3, Table S7, Supporting Information).^[^ ^32^, ^33^, ^34^ ^]^ Likewise, ZARs become more selective for serotonin over dopamine in the presence of salts (selectivity ratio of 17 in water versus 40 in phosphate buffer, Figure 5f–h and Table S4 and S7, Supporting Information).

### 2.4 ZAR‐Based Sensing in Biofluids

Abnormal concentration levels of neurotransmitters are both the cause and indicators for neurological disorders, such as depression and migraine. Furthermore, they serve as markers for a wide variety of body malfunctions, for example, tumors and neurodegenerative diseases, and are linked to the irritable bowel syndrome.^[^ ^54^, ^58^, ^59^, ^60^ ^]^ Shortcomings of current diagnostic methods, unfortunately, hamper the routine neurotransmitter sensing for disease diagnosis and treatment. Monoclonal antibodies have become the “gold standard” but lack a direct signal response and often require long assaying times.^[^ ^48^ ^]^ Conversely, neurotransmitter‐binding to natural receptor proteins and fluorescent mutants is rapid,^[^ ^46^, ^47^, ^61^ ^]^ but their large‐scale preparation is expensive and their practical application requires specialized laboratories.

**Figure 6:**
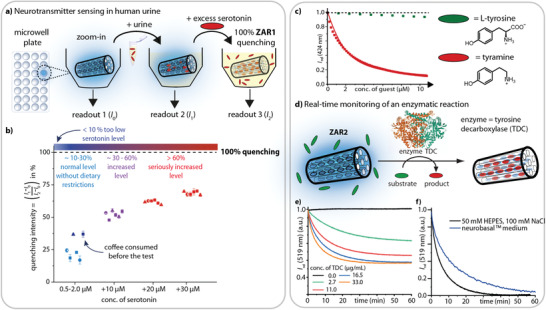
Applications of ZARs. a) Schematic presentation of a facile ZAR‐based neurotransmitter detection assay in human urine. After first readout with **ZAR1** in aq. dispersion (*I*_0_), human urine is added and the signal detected (*I*_1_). Subsequently, an excess of serotonin is added, and the residual background emission is recorded (*I*_2_). b) Experimental results for serotonin levels in spot urine samples from healthy volunteers (partly spiked, see x‐axis). These spiked urine samples, corresponding to typical serotonin levels for cancer patients, can be clearly identified. (High accuracy and precision of the assay are confirmed for samples with known serotonin concentrations in synthetic urine; typical serotonin levels found for clinically depressed subjects can be reliably quantified, Figure S19–S22, Supporting Information.) c) Binding titration curve for the weakly binding L‐tyrosine (green) and its strongly binding decarboxylation product tyramine (red). d) Schematic depiction of the decarboxylation of L‐tyrosine to tyramine by tyrosine decarboxylase (TDC) enzyme with **ZAR2**. e) Real time monitoring of tyrosine turn‐over with different TDC concentrations (0–33 µg mL^−1^). f) Real time monitoring of TDC‐catalyzed tyrosine decarboxylation is possible in highly complex reaction media such as neurobasal^TM^ medium, which is infeasible with known colorimetric or supramolecular tandem assays.

The affinities and selectivity of ZARs for neurotransmitters in saline aqueous media are so high that we were eager to evaluate their performance in complex biological media (Figure S17–S18, Supporting Information). Indeed, an excellent performance of ZARs for serotonin and dopamine detection and binding (*K*_d_(serotonin) = 1.0 µM and *K*_d_(dopamine) = 48.0 µM) was obtained in neurobasal^TM^ medium composed of 37 different organic components and salts, and which is used to maintain neurons in culture. ZARs retained their micromolar affinities for neurotransmitters in urine (*K*_d_(serotonin) = 2.6 µM and *K*_d_(dopamine) = 7.8 µM) and human blood serum (*K*_d_(serotonin) = 1.3 µM and *K*_d_(dopamine) = 4.2 µM), where diagnostic assays are performed. It is of medical relevance to identify patients that either show abnormally high (>10 µM; tumor indicator) or abnormally low (≤0.8 µM; indicator for neurological disorders) neurotransmitter levels. Therefore, we tested the potential of ZARs for diagnostic applications in urine, which is a biofluid that can be conveniently and regularly obtained, even by the layman. Indeed, through the ZAR‐based assay, urine samples from healthy volunteers can be readily distinguished from those with artificially modified abnormally high levels of serotonin (**Figure** **6**a,b). Likewise, samples with abnormally low serotonin concentrations, ≤0.8 µM, are identifiable (Figure S19–S22, Supporting Information). Noteworthy, we found that additional spiking of urine with dopamine did not significantly affect the serotonin detection in urine samples (up to 22 µM dopamine was added to simulate abnormally high dopamine levels^[^ ^62^ ^]^), pointing to the aforementioned salt‐induced selectivity enhancement of ZARs.

As the ZAR‐based assay does not require sample pre‐treatment or washing steps and can be carried out in cheap microwell plates with fluorescence plate readers, we are confident that it will be applicable in clinics and point‐of‐care units. We also believe that ZAR‐based sensors for home‐use can be developed, which could support individualized therapies^[^ ^63^ ^]^ by providing information about the drug dose influence on the physiological neurotransmitter levels.

### 2.5 Label‐Free Enzymatic Reaction Monitoring

We discovered, that besides detecting and quantifying neurotransmitters in a diagnostic assay, ZARs can be used for label‐free enzymatic reaction monitoring in real time. For comparison, this is a great practical challenge with existing technologies that provide only discontinuous data points and require time‐consuming sample pre‐ and post‐treatment steps. The ZAR‐based strategy allows for the detection and quantification of enzymatic reaction products (or substrates) also for medically relevant purposes. For example, tyrosine decarboxylase (TDC) enzyme is expressed by bacteria in the human gut, and causes the frequently witnessed ineffectiveness of oral l‐DOPA administration for Parkinson's disease treatment.^[^ ^64^, ^65^ ^]^ Improving on SB‐based tandem assay concepts,^[^ ^66^, ^67^ ^]^ it is now possible with ZARs to monitor the enzymatic reaction of TDC in real time in “biological buffers” and in complex biofluids (Figure 6 and Figure S27–S29, Supporting Information). Briefly, L‐tyrosine is decarboxylated by TDC to yield tyramine as product which is readily captured by ZARs. This approach can be extended to other enzymes that play a role in biosynthesis or catabolism of neurotransmitters and can be adopted to widen the scope of target analytes, for example, to L‐tryptophan or L‐phenylalanine and their derivatives, through exploiting additional enzyme‐ZAR tandem assays.

### 2.6 Generalizability and Scope of ZAR Design

**Figure 7:**
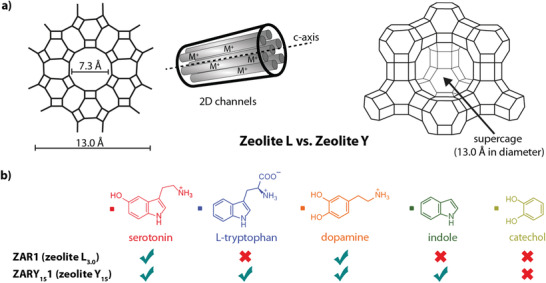
Generalizability and scope of ZARs. a) Schematic representation of the zeolite L and zeolite Y framework. b) Comparison of analyte binding scope for ZARs that are obtained by loading indicator dye D1 into the micropores of zeolite L and zeolite Y.

The modular and tunable ZAR design opens up additional sensing possibilities for other metabolites and small bioactive target molecules, particularly for hydrophilic analytes. For instance, the electron‐poor neurotransmitter histamine can be detected and distinguished from dopamine in both emission switch‐on and ratiometric assays by ZARs with self‐aggregating reporter dyes (Figures S36 and S37, Supporting Information). Furthermore, different framework materials, for example, zeolite Y, can be used to tune the binding affinities and selectivity. Even non‐charged aromatic and zwitterionic compounds such as indole and tryptophan can be bound and detected by ZARs based on dealuminated and thus more hydrophobic zeolite Y_15_, with a Si‐to‐Al ratio of 15 (Figure S38–S40, Supporting Information), while a clear binding preference for positively charged neurotransmitters is found for zeolite L‐based ZARs with a Si‐to‐Al ratio of 3 (**Figure** **7**). In this way, many different ZARs can be readily prepared and applied to array‐based sensing formats, where the combination of confinement and electronic dye‐analyte interactions will allow the differentiation amongst even structurally similar target molecules.

## 3 Conclusion

We have demonstrated that the rational combination of microporous inorganic materials with tailor‐made reporter dyes provides a promising platform for the design of artificial receptors for neurotransmitters. The herein introduced ZAR design concept is modular and facile and can be used to prepare powerful sensor materials at a gram scale. ZARs outperform all known SBs for neurotransmitter sensing by orders of magnitude in terms of affinity and selectivity. Compared to biobased receptors, for example, antibodies, ZARs are much faster responding, chemically and thermally much more robust, cost‐economic, and show direct signal transduction capabilities. As such, ZARs can be used for monitoring of biophysical processes, for example, for label‐free enzymatic reaction monitoring and for (two‐photon) imaging of neurotransmitters. The modular design capabilities will enable tuning of binding affinity and selectivity towards other metabolites, neurotransmitters, and hormones and can provide the base for developing rapid diagnostic tests for home‐use and point‐of‐care applications. We believe that the ZAR materials design concept can strongly impact metabolomics, theranostics, and fundamental medical/biological research.

## 4 Experimental Section

### Synthesis of 2,7‐Dimethylbenzo[lmn][3,8]phenanthroline‐2,7‐diium Diiodide (D1)^[^ ^68^ ^]^

2,7‐Diazapyrene was synthesized starting from 1,4,5,8‐naphthalenetetracarboxylic dianhydride in a three‐steps synthesis following literature procedures.^[^ ^69^, ^70^ ^]^ Under nitrogen atmosphere, 2,7‐diazapyrene (97.0 mg, 475 µmol, 1.0 eq) was dissolved in dry DMF (12 mL). Methyl iodide (1.00 mL, 2.28 g, 16.1 mmol, 34.0 eq) was added and the reaction solution was stirred at room temperature overnight. Afterwards, another portion of methyl iodide (1.00 mL, 2.28 g, 16.1 mmol, 34.0 eq) was added and the reaction solution was stirred at room temperature overnight. The yellow precipitate was filtered off, washed with DMF, and recrystallized from methanol. The crude product was dissolved in 1 M HCl, overlaid with acetone and the mixture was stored at 4 °C overnight. The precipitate was filtered off and washed with acetone. The product (**D1**) was isolated as a yellow solid with a yield of 65% (151 mg, 309 µmol). ^1^H NMR (500 MHz, D_2_O, δ): 10.03 (s, 4H, *H*‐Ar), 8.85 (s, 4H, *H*‐Ar), 4.97 (s, 6H, CH_3_) ppm; ^13^C NMR (126 MHz, D_2_O, δ): 141.9 (CH), 129.9 (CH), 129.5 (C_q_), 126.8 (C_q_), 49.9 (CH_3_) ppm; ESI‐MS *m/z* calcd. for C_16_H_14_N_2_^2+^ 117.0573, found 117.0569.

### Synthesis of 2,9‐Dibenzylanthra[2,1,9‐6,5,10]diisoquinoline‐2,9‐diium Dichloride (**D2**)

2,9‐Dibenzyl‐1,2,3,8,9,10‐hexahydroanthra[2,1,9,6,5,10]diisoquinoline was synthesized following literature procedures.^[^ ^69^, ^70^ ^]^ A 100 mL round bottom flask with connected reflux condenser was charged with the diquinoline (1.00 g, 1.94 mmol, 1.0 eq) and 2,3‐dichloro‐5,6‐dicyano‐1,4‐benzoquinone (DDQ) (3.20 g, 14.0 mmol, 7.2 eq). Afterwards, dry acetonitrile (100 mL) was added, and the mixture was stirred overnight at room temperature and then another 7 days at reflux. The solution was cooled to room temperature and concentrated HCl (5 mL) and acetone (150 mL) were added. A red precipitate formed, and the solution was stored at 4 °C overnight. The solid was collected by filtration and washed with acetone. The solid was dissolved in 1 M HCl (20 mL) and filtered. Upon addition of acetone (300 mL), a red precipitate formed. The flask was left in the fridge overnight and the solid was collected by filtration and washed with acetone. The product (**D2**) was isolated with a yield of 27% (350 mg, 524 µmol). ^1^H NMR (400 MHz, D_2_O, δ): 9.57 (s, 4H), 8.10‐7.55 (m, 18H), 6.25 (s, 4H) ppm; ^13^C NMR (101 MHz, D_2_O, δ): 159.2 (CH), 137.5 (C_q_), 133.8 (CH), 133.7 (C_q_), 130.8 (CH), 130.2 (CH), 129.7 (C_q_), 125.6 (CH), 65.8 (CH_2_) ppm (Note: Due to low solubility and stacking, not all quaternary carbons appear after 10 000 scans.); IR (ATR): ν = 3036 (m), 3007 (m), 2933 (m), 2882 (m), 2815 (m), 2755 (m), 1455 (m), 1405 (m), 1381 (m), 1365 (m), 1340 (m), 1281 (m), 1268 (m), 1135 (m), 1108 (m), 892 (m), 821 (s), 761 (m), 737 (m), 701 (m) cm^−1^; ESI‐MS *m*/*z* calcd. for C_38_H_26_N_2_^2+^ 255.1043; found 255.1041.

### Preparation of the ZARs

A dicationic dye was solubilized in 10 mL deionized water and the stock solution concentration was determined by extinction coefficient‐based absorbance measurements (**D1**: λ = 393 nm, ε = 7800 M^−1^ cm^−1^; **D2**: λ = 431 nm, ε = 26 000 M^−1^ cm^−1^). Precisely weighed in zeolite powder was added to the solution. After 10 min of treatment with a tip sonicator (up200s hielscher, working frequency 30 ± 1 kHz, energy density ≥300 W cm^2^), the suspensions were centrifuged, decanted, and washed copiously with water to remove surface‐physiosorbed dye molecules. This sequence was repeated until the supernatant became colorless and non‐emissive. Generally, after the second washing cycle, no quantifiable amounts of unbound dye remained. Dye loading was found to be possible in a range of 0–4 wt% (wt% based on the amount of zeolite used). Finally, the solids were dried in vacuum to yield the corresponding ZARs. Different ZARs with loadings in the range of 0.23–2.3 wt% per weight were prepared. The loading was generally controlled by precise weighing on high precision laboratory balances and verified by absorbance and emission measurements as well as elemental analysis (average value of the carbon‐based quantifications).

### Photophysical Experiments

Absorption spectra were measured either on a shimadzu UV‐3600 double‐beam UV–Vis–NIR spectrophotometer or on a jasco V‐730 double‐beam UV–Vis spectrophotometer and baseline corrected. Solid‐state UV–Vis spectra were recorded against a background of BaSO_4_ and normalized to equal absorbances at 425 nm (**D1**) or 420 nm (**D2**). All UV–Vis spectra were recorded with 2.3 wt% dye loading. Relative absorbance plots were referenced to the isosbestic point at 425 nm. Steady‐state emission spectra were recorded either on a horiba jobin−yvon IBH FL‐322 Fluorolog 3 spectrometer equipped with a 450 W xenon arc lamp, double‐grating excitation, and emission monochromators and a TBX‐04 single‐photon counting detector or on a jasco FP‐8300 fluorescence spectrometer equipped with a 450 W xenon arc lamp, double‐grating excitation and emission monochromators. Emission spectra were corrected for source intensity (lamp and grating) and emission spectral response (detector and grating) by standard correction curves. Detailed information on the concentrations of zeolite and dye loading is listed in Table S5, Supporting Information, for each individual measurement. Microplate assays were performed on a perkin elmer EnSight multimode plate reader in OptiPlate‐96 microplates. Quantum yield measurements were performed on a horiba jobin‐yvon IBH FL‐322 Fluorolog‐3 spectrometer with a Quanta‐φ integrating sphere as an accessory attached. The data was acquired by the commercially available software FluorEssence (horiba jobin‐yvon) version 3.5. Stopped‐flow experiments were carried out on a jasco FP‐8300 fluorescence spectrometer equipped with a water thermostated (*T* = 25 °C) SFA‐20 stopped‐flow accessory from tgk scientific limited, which was driven by a pneumatic drive.

### Confocal Fluorescence Microscopy

Imaging was performed on an abberior easy3D STED/RESOLFT QUAD scanning microscope (abberior instruments) built on a motorized inverted microscopeIX83 (olympus) and equipped with a 100×/1.40 UPlanSApo oil immersion objective lens (olympus). Particles were excited with a pulsed laser at 485 nm and detection was performed with avalanche photodiodes (APD) in a spectral window from 505 to 725 nm. Images were smoothed with a 1‐pixel lowpass Gaussian filter. Quantification of the fluorescence intensity of the individual particles was performed on the raw images in ImageJ 1.50b with background subtraction and normalized to the respective particle intensity before dopamine application or at frame 1. 155 and 20 particles were analyzed to quantify the fluorescence recovery after dopamine removal and during dopamine application, respectively.

### Isothermal Titration Calorimetry (ITC)

ITC experiments were carried out on a Microcal PEAQ‐ITC from malvern panalytics at 25 °C. Aqueous zeolite or ZAR dispersions were filtered with a 0.45 µm syringe filter (polypropylene) prior to the experiments and added into the cell of the instrument, taking care that no air bubbles remained. Similarly, the aqueous solutions of the synthetic binders were placed into the cell. The same concentration of ZARs (calculated with respect to number of binding sites, that is, molar concentration of dye), zeolite L (same wt% as for corresponding ZARs), and SBs (same molar concentration of binding sites) was used in each experiment. In a typical experiment, 1.5 µL titrant solution (the first injection was 0.4 µL) with 150 s spacing and 5 μcal s^−1^ reference power were injected 25 times into the ITC cell (stir speed: 750 rpm; initial delay: 60 s; injection duration: 6 s). The raw data was analyzed by Microcal PEAQ‐ITC analysis software using a 1:1 complexation model and the first data point was always omitted. All data were baseline corrected by the average value of the titration curve into water and corrected by an additional offset due to buffer mismatch effects caused by the ion leaching from the zeolites and ZARs.

### Ethical Approval and Informed Consent

All procedures performed in studies involving human participants were in accordance with the formal statement of ethical principles published by the World Medical Association in the declaration of Helsinki in 1964 and its later amendments or comparable ethical standards.^[^ ^71^, ^72^, ^73^ ^]^ Informed consent was obtained from all individual participants included in the study.^[^ ^74^, ^75^ ^]^

## Conflict of Interest

The authors declare no conflict of interest.

## Supporting information

Supporting Information

## Data Availability

The data that support the findings of this study are available from the corresponding author upon reasonable request. Synthetic data on dye synthesis are openly available in Chemotion repository at https://www.chemotion-repository.net. Binding data of SB‐neurotransmitter complexes can be found found in SupraBank at https://SupraBank.org.
