# Supplementary material for: Fluorescent Nanozeolite Receptors for the Highly Selective and Sensitive Detection of Neurotransmitters in Water and Biofluids
Source: Adv Mater. 2021 Sep 27;33(49):2104614. doi: 10.1002/adma.202104614 (PMC11468822; doi:10.1002/adma.202104614)
Supplement: Supplementary file 1 — Supporting Information [file ADMA-33-2104614-s001.pdf]

© 2021 Wiley-VCH GmbH

ADVANCED MATERIALS

Supporting Information

for *Adv. Mater.,* DOI: 10.1002/adma.202104614

# Fluorescent Nanozeolite Receptors for the Highly Selective and Sensitive Detection of Neurotransmitters in Water and Biofluids

*Laura M. Grimm, Stephan Sinn, Marjan Krstić, Elisa D'Este, Ivo Sonntag, Eko Adi Prasetyanto, Thomas Kuner, Wolfgang Wenzel, Luisa De Cola,* and Frank Biedermann**

Supporting Information for

**Fluorescent nanozeolite receptors for the highly selective and sensitive detection of neurotransmitters in water and biofluids**

*Laura M. Grimm, Stephan Sinn, Marjan Krstić, Elisa D'Este, Ivo Sonntag, Eko Adi Prasetyanto, Thomas Kuner, Wolfgang Wenzel, Luisa De Cola and Frank Biedermann*

Correspondence to: luisa.decola@marionegri.it & frank.biedermann@kit.edu

## Table of Content

1. Abbreviations ... 2
2. Materials and methods ... 3
2.1. Equipment ... 3
2.2. Materials preparation ... 4
2.3. Materials characterization ... 8
2.4. Data statement ... 8
3. Discussion of the binding mechanism ... 9
3.1. Data analysis and extraction of the dissociation constants $K_d$ ... 12
4. Comparison between bioreceptors and artificial binders ... 13
5. Photophysical properties ... 15
5.1. Excitation and emission spectra of ZARs ... 15
5.2. Fluorescence titration experiments and binding curves ... 16
5.3. Ratiometric sensing experiments ... 20
5.4. Absorbance-based measurements ... 22
5.5. Two-photon microcopy ... 24
6. Selectivity ... 24
7. Fluorescence titration experiments in biological relevant media ... 28
8. Assays in synthetic urine (surine) and human urine ... 30
9. Thermodynamic characterization of the ZAR-analyte binding process ... 33
10. Monitoring of label-free enzymatic reactions in real time ... 35
11. Functionality of ZARs in organic solvents ... 37
12. Theoretical methods ... 38
13. Further scope of ZARs ... 42
13.1. Detection of non-quenching neurotransmitters, *e.g.*, histamine ... 42
13.2. Fluorescence turn-on assays ... 43
13.3. Detection of neutral and zwitterionic analytes ... 44

14. Synthesis ........ 46
15. References ........ 47

## 1. Abbreviations

| | |
|---|---|
| A | Analyte |
| AO | Atomic orbital |
| AA | Ascorbic acid |
| AR | Adrenergic receptor |
| ATR | Attenuator total reflection |
| $c_B$ | Nu[illegible]ber of binding sites |
| $C^d$ | Dye decorated cavity |
| $C^dG$ | Complex of bound guest towards a dye decorated cavity |
| $C^e$ | Empty cavity |
| $C^eG$ | Complex of bound guest towards an empty cavity |
| CE | Cellulose ester |
| CHO | Chinese hamster ovary |
| conc. | Concentration |
| CT | Charge transfer |
| D | (indicator / reporter) dye |
| DAP | 2,7-Diazapyrene |
| DEPT | Distortionless enhancement by Polarization transfer |
| DLS | Dynamic light scattering |
| EM | Electron microscopy |
| ELISA | Enzyme-linked immunosorbent assay |
| eq | Equation |
| $F_0$ | Emission intensity before analyte addition |
| $F_A$ | Intensity at a given analyte concentration |
| HEPES | (4-(2-Hydroxyethyl)-1-piperazineethanesulfonic acid) |
| HS | Human serum (human male AB plasma) |
| HSA | Human serum albumin |
| IR | Infrared |
| ITC | Isothermal titration calorimetry |
| $K_a$ | Association constant |
| $K_d$ | Dissociation constant |
| PBE | Perdew-Burke-Ernzerhof |
| PBS | Phosphate buffered saline |
| ppm | Parts per million |
| PRP | Pyridoxal 5’-phosphate hydrate |
| RI | Resolution of identity |
| SB | Synthetic binder |
| SEM | Scanning electron microscopy |
| T | Temperature |

| | |
|---|---|
| TD | Time-dependent |
| TDC | Tyrosine decarboxylase |
| wt% | Weight percentage |
| ZAR | zeolite-based artificial receptor |
| $\Delta F$ | Relative emission increase or decrease caused by analyte addition |
| $\tau$ | Diffusion coefficient |
| $\lambda_{em}$ | Emission wavelength |
| $\lambda_{ex}$ | Excitation wavelength |

# 2. Materials and methods

## 2.1. Equipment

Nuclear magnetic resonance (NMR) spectroscopy

NMR spectra were recorded on a BRUKER Avance 500 ($^{1}$H NMR: 500 MHz; $^{13}$C NMR: 126 MHz) at room temperature. The chemical shift $\delta$ is expressed in parts per million (ppm) whereas the residual signal of the solvent has been used as secondary reference: dimethylsulf-oxid-$d_6$ ($^{1}$H: $\delta$ = 2.50 ppm, $^{13}$C: $\delta$ = 39.5 ppm), $D_2O$ ($^{1}$H: $\delta$ = 4.90 ppm).[1,2] $^{1}$H NMR spectra were analyzed according to first order, $^{13}$C spectra were $^{1}$H-decoupled and characterization of the $^{13}$C NMR spectra was ensued through the DEPT-technique (DEPT = distortionless enhancement by polarization transfer) and is stated as follows: DEPT: "+" = primary or secondary carbon atoms (positive DEPT-signal), "-" = secondary carbon atoms (negative DEPT-signal), $C_q$ = quaternary carbon atoms (no DEPT-signal). For central symmetrical signals, the midpoint is given, for multiplets the range of the signal region is given.

Infrared spectroscopy (IR)

IR spectra were recorded on a THERMO SCIENTIFIC™ Nicolet™ iS™50 FTIR spectrometer with a built-in attenuator total reflection (ATR) module. Measurements of the samples were conducted via ATR and were measured in the range from 4000 cm$^{-1}$ to 400 cm$^{-1}$. The band intensity (strength of absorption) was described as follows: vs = very strong (0 - 9.9% trans-

mission T); s = strong (10 - 39.9% T); m = middle (40 - 69.9% T); w = weak (70 - 89.9% T); vw = very weak (90 - 100% T). The position of the absorption bands is given as wavenumber $\tilde{\nu}$ with the unit ($cm^{-1}$).

Mass spectrometry (ESI-MS)

Electrospray ionization (ESI) mass spectrometry experiments were carried out on a BRUKER micrOTOF-Q (208 - 320 Vac, 50/60 Hz, 1800 VA) mass spectrometer equipped with an ON-LINE NANOELECTROSPRAY ion source. The spectra were interpreted by molecular peaks $[M]^{n+}$, peaks of protonated molecules $[M+H]^{n+}$ and characteristic fragment peaks and indicated with their mass-to-charge ratio (m/z). Solvents used were $H_2O$, MeOH and DMSO.

Confocal fluorescence microscopy

For sample preparation, **ZAR2** particles (250 µg $mL^{-1}$; 2.3 wt% dye loading) were sonicated for 25 min in a water bath at room temperature, diluted 1:6 in $H_2O$, incubated on a polylysine coated coverslip for 20 min, and washed prior to imaging in $H_2O$. Dopamine was added to the sample at a final concentration of 50 µM and time-lapse imaging was started immediately afterwards. Rinsing was performed by exchanging the medium with water.

Two-photon microscopy

**ZAR1** particles were immobilized on a glass coverslip using fibroin gel (SIGMA ALDRICH #5154), superfused with PBS or PBS containing different dopamine concentrations, and excited through a water immersion objective at a wavelength of 800 nm using a TriM Scope II microscope (LAVISION BIOTEC GMBH) equipped with a pulsed Ti:Saphire laser (Chameleon Ultra II; COHERENT).[3]

### 2.2. Materials preparation

Solvents and reagents

The chemicals for the synthesis were purchased from MERCK, SIGMA ALDRICH, ACROS ORGANICS and ALFA AESAR with the minimum quality "for synthesis" and were used without further purification. Dry solvents were stored over molecular sieve (3Å or 4Å) to ensure their aridity over long periods. Analytes were purchased from SIGMA ALDRICH, TCI and ALFA AESAR with the highest purity grade available, typically as analytical standard grade and used as received. The zeolite $L_{3.0}$ host (Lucidot® NZL 40) was provided by CLARIANT,[4] zeolite $Y_{2.55}$ was purchased as its sodium salt from ALFA AESAR and zeolite $Y_{15}$ was purchased from ZEOLYST INTERNATIONAL as its $H^+$ form (CBV720), see **Table S1**.

**Table S1.** Properties of zeolite samples that were utilized as cavity-framework materials for the assembly of ZARs. Information about the formula of unit cells is given as from the supplier received;[5,6] water molecule numbers were taken from ref. [7] for LTL and from ref. [8] for FAU.

| **Zeolite class** | **Linde-type (LTL, L)** | **Faujasite (FAU, Y)** | |
|---|---|---|---|
| **Supplier code** | Clariant, Lucidot® NZL 40 | Alfa Aesar 45862 | Zeolyst CBV720 |
| **Connectivity** | 2D channels | 3D network | 3D network |
| **Formular of unit cell (hydrated)** | $\|K^+_6Na^+_3\|[Al_9Si_{27}O_{72}] \cdot \sim 20\ H_2O$ | $\|Na^+_{54}\|[Al_{54}Si_{138}O_{384}] \cdot \sim 240\ H_2O$ | $\|Na^+_{12}\|[Al_{12}Si_{180}O_{384}] \cdot \sim 240\ H_2O$ |
| **max. diameter of sphere that can be included (Å)**[6] | 10.0 | 11.3 | 11.3 |
| **max. diameter of sphere that can diffuse along (Å)**[6] | 7.5 | 7.35 | 7.35 |
| **Si/Al ratio** | 3.00 | 2.55 | 15.0 |

Ion exchange within the zeolite pores[9-11] was conducted to displace hydrogen cations by sodium cations. For this purpose, the zeolite dispersions were sonicated with a copious amount of $NaHCO_3$. Afterwards, the mixtures were centrifuged and washed four times with water. Dialysis membranes, namely Spectrum™ Spectra/Por™ Biotech Cellulose Ester (CE) dialysis membrane tubing with a MWCO from 100 to 500 D, were purchased from FISHER SCIENTIFIC.

Analytes were purchased from SIGMA ALDRICH, TCI and ALFA AESAR with the highest purity grade available, typically as analytical standard grade and used as received. Human serum (HS) was purchased as human serum from human male AB plasma provided by SIGMA, whereas fatty acid free human serum albumin (HSA) protein was purchased from ALFA AESAR. Neuro-basal™ medium (minus phenol red) was purchased from THERMO FISHER SCIENTIFIC. Surine was purchased from CERILLIANT. For the human urine, healthy coworkers voluntarily donated their own urine, which was spiked with serotonin and/or dopamine to simulate diseases that are indicated by high neurotransmitter levels. No health-relevant information about the non-spiked urine was gathered. TDC was purchased from CREATIVE ENZYMES as native *streptococcus faecalis* L-tyrosine decarboxylase. Buffer solutions were prepared following standard protocols. For 1X PBS, buffer tablets from CRUZ CHEM were dissolved in 500 mL MilliQ water.

Preparation of the ZARs

Different ZARs with loadings in the range of 0.23 wt% - 2.3 wt% per weight were prepared. The loading was in general controlled by precise weighing on high precision laboratory balances and verified by absorbance and emission measurements as well as elemental analysis (average value of the carbon-based quantifications). Absorbance measurements were utilized to quantify the dye amount inside the supernatant of the sonicated and centrifuged suspensions. This allowed the acquisition of the residual dye concentration in the solutions and thus the corresponding loading inside the zeolite particles by subtracting from the originally added dye concentration. For example, 2.3 wt% of **D1** were loaded in zeolite $L_{3.0}$ to form **ZAR1**, a dye concentration of 2.3 wt% was confirmed by absorbance measurements, while the elemental analysis showed a value of 2.4 wt%. Similar results were found for a dye loading of 0.23 wt%, which equals a **D1** concentration of 1.63 µM for a **ZAR1** zeolite concentration of 250 µg $mL^{-1}$. The obtained dye concentration (ZAR loading) values are in

good agreement with the independently determined inflection point of the fluorescence-based binding isotherms (applying a 1:1 binding model) of ZARs with a strong binder such as serotonin. At the inflection point, the concentration of fluorescent binding cavities is equal to the titrant concentration. Detailed information on the concentrations of zeolite and dye (loading) is listed in **Table S5** for each individual measurement.

Preparation of ZAR stock solutions in water, buffers and biofluids

ZAR dispersions in buffered or synthetic biological media were prepared by weighing in the ZAR solid (three significant figures) to which the accurately measured volume of the medium was added. The mixture was then sonicated by a tip sonicator (UPS HIELSCHER, working frequency 30 kHz, energy density ≥ 300 W $cm^{-2}$) for 10 min and used as such.

For neurobasal™ medium, **ZAR2** was directly dispersed in the medium and used as such. For human serum albumin (HSA) measurements, **ZAR2** was dispersed in 50 mM HEPES, pH 7.2, and sonicated with HSA dissolved in 50 mM HEPES, pH 7.2 ($c_{end}$(HSA) = 250 µM). For human serum (HS), **ZAR2** was dispersed in 50 mM HEPES, pH 7.2, and sonicated 2:1 (v/v) with HS. For surine, **ZAR2** was dispersed in 50 mM HEPES, pH 7.2, and sonicated 1:1 (v/v) with surine.

Preparation of analyte stock-solutions

The concentration of the analyte stock solution, typically 100 µM to 10 mM, was adjusted that less than 10% dilution occurred upon addition of 3 - 10 equivalents of the analyte (as compared to the concentration of the dye in the ZAR) to the cuvette containing the ZAR suspension. The analyte concentrations were independently assessed by their absorbances, utilizing reported extinction coefficients. It was confirmed that the intended concentrations by weighing in (3 significant figures) and the actual concentration of the analyte stock solution where always within 5% error.

### 2.3. Materials characterization

**Table S2.** Zeta potential of the used zeolites and their ZARs in MilliQ water or 1X PBS and their average diameter size (size distribution by intensity), determined by dynamic light scattering (DLS). Errors in DLS ≤ 40%. Filtration with a MillexGS 0.22 µM PS syringe filter prior to the measurements did not alter the results and therefore the results are not separately given.

| Analyte | Zeta potential in MilliQ water (mV) | Zeta potential in 1X PBS (mV) | Averaged diameter size (nm) |
|---|---|---|---|
| **Zeolite $L_{3.0}$** | –30 | –32 | 180 |
| **ZAR1** | –27 | –34 | 180 |
| **ZAR2** | n.d. | –28 | 180 |
| **Zeolite $Y_{2.55}$** | n.d. | –27 | 1800 |
| **$ZARY_{2.55}1$** | n.d. | –31 | 1800 |
| **$ZARY_{2.55}2$** | n.d. | –28 | 1800 |
| **Zeolite $Y_{15}$** | –38 | n.d. | 700 |
| **$ZARY_{15}1$** | –36 | n.d. | 700 |

n.d. = not determined

### 2.4. Data statement

Statistics and reproducibility

Dissociation constants were determined at least in triplicates. Average values (mean) and their standard deviation are shown in this work. The experiments were replicated by different operators over the course of five years, using different batches of ZARs and different instrument models from different producers. Within the margin of error, the results were found to be highly reproducible.

Data availability

All data are available from the corresponding authors upon reasonable request and are digitally stored on the servers of the home institution. Synthetic procedures and characterization data of the indicator dyes and precursors can be found at the Chemotion

repository, https://www.chemotion-repository.net/. Furthermore, binding parameters can be found on the SupraBank repository, https://suprabank.org/.

## 3. Discussion of the binding mechanism

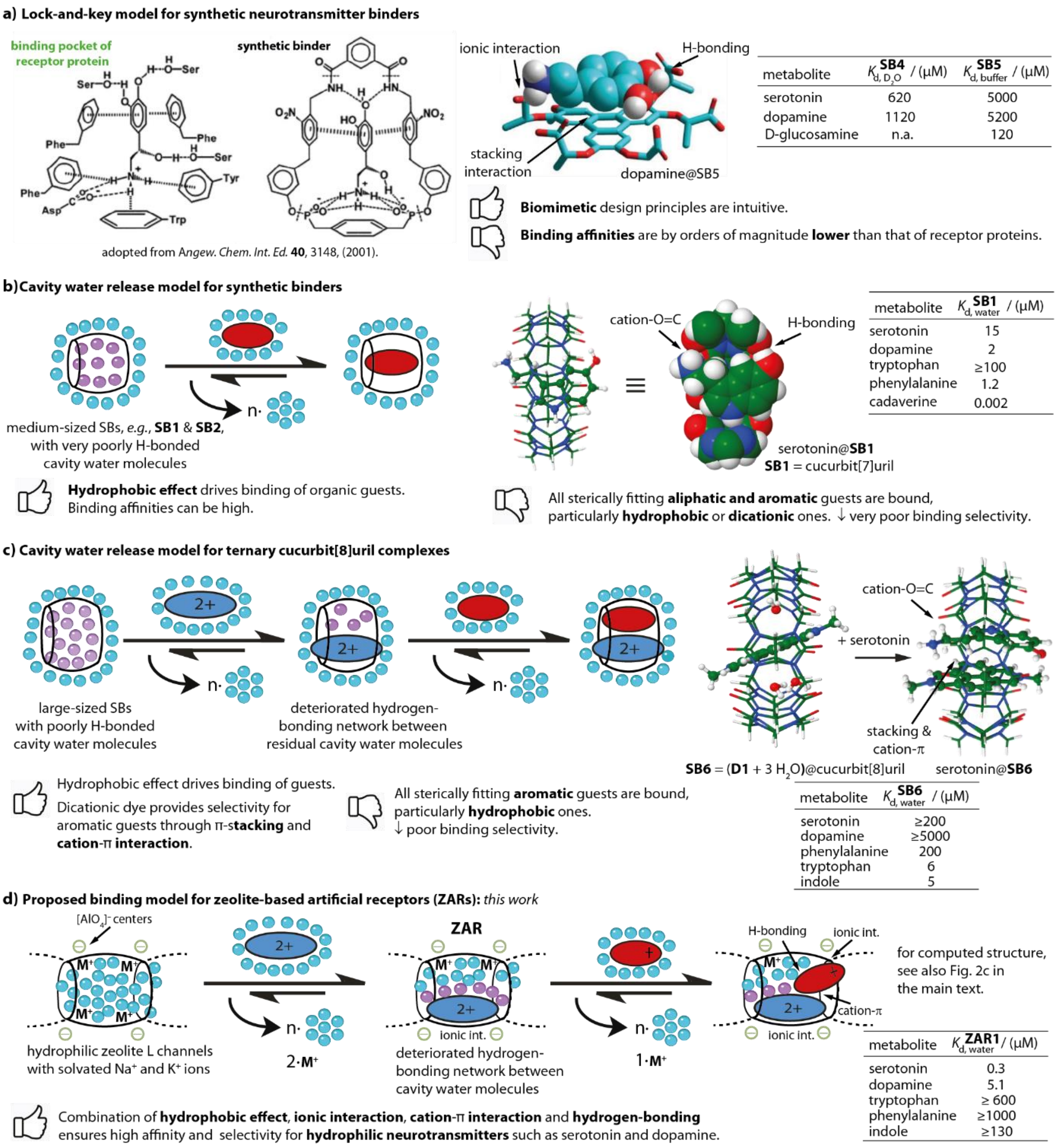


**Fig. S1.** Cavity water release models and proposed binding mechanism for artificial receptors. Further explanation in the text.

Synthetic binders designed to recognize neurotransmitters (NTs) through direct non-covalent binding motifs, *e.g.*, salt bridges and stacking interactions, can be relatively selective for their target molecules but provide impractically low binding affinities. **Figure S1a** shows a binding pocket of a receptor protein (β-adrenergic receptor) for norepinephrine and the lock-and-key design of a synthetic NT binder.[12]

In another approach, the cavity water release model for synthetic binders is utilized. Medium-sized SBs such as **SB1** contain poorly H-bonded cavity water molecules that are released upon binding of aliphatic and aromatic guest molecules, and thereby restore their hydrogen bonding potential (**Figure S1b**). This contributes an exothermic driving force to the binding event, which is referred to as the “non-classical hydrophobic effect”.[13] However, despite high binding affinities only poor binding selectivity is reached by such a binding mechanism. For bigger SBs such as cucurbit[8]uril (CB8), the formation of ternary complexes is driven by the hydrophobic effect (cavity water release)[13] and by cation-π- and π-π-interactions (**Figure S1c**). Higher selectivity is reached than for purely cavity water release driven SBs, but these systems cannot be used for serotonin/dopamine detection in biofluids because they preferentially bind more hydrophobic amino acids such as L-phenylalanine and L-tryptophan, and many other aromatic species.

The within this manuscript proposed binding model for zeolite-based fluorescent artificial receptors (ZARs) is shown in **Figure S1d**. Cations bound inside the negative zeolite framework can be replaced by positively charged dye molecules such as **D1** and **D2**. As a result of the interplay of hydrophobic effect, ionic interactions, cation-π-interactions and hydrogen bonding, very high binding affinities *and* selectivity for hydrophilic neurotransmitters such as serotonin are reached.

For the interaction of a guest with pure zeolite, uniform binding sites are assumed and thus a 1:1 binding model was applied:

$$C^e + \mathrm{G} \rightleftarrows C^e\mathrm{G} \quad \text{Eq. S1}$$

$$K_\mathrm{d}^\mathrm{e} = \frac{[C^e][\mathrm{G}]}{[C^e\mathrm{G}]} \quad \text{Eq. S2}$$

where $C^e$ denotes an empty cavity, G denotes the free guest, $C^e$G denotes the complex of bound guest towards an empty cavity and $K_d$ the appropriate dissociation constant.

For the interaction of a guest (analyte) with a ZAR, two different binding sites are available at the host (ZAR): (*i*) binding towards an empty cavity, (*ii*) binding towards a dye decorated cavity. These possibilities imply a competitive binding model where two binding sites compete for one guest:

$$C^e + \mathrm{G} \rightleftarrows C^e\mathrm{G} \qquad C^c + \mathrm{G} \rightleftarrows C^d\mathrm{G} \quad \text{Eq. S3}$$

$$K_\mathrm{d}^\mathrm{e} = \frac{[C^e][\mathrm{G}]}{[C^e\mathrm{G}]} \qquad K_\mathrm{d}^\mathrm{d} = \frac{[C^d][\mathrm{G}]}{[C^d\mathrm{G}]} \quad \text{Eq. S4}$$

where $C^e$ denotes an empty cavity binding site, $C^d$ denotes a dye decorated cavity, G denotes the free guest, $C^e$G denotes the complex of bound guest towards an empty cavity, $C^d$G denotes the complex of bound guest towards a dye decorated cavity and $K_d$ the appropriate dissociation constant.

The determined binding affinities of the analytes towards the pure zeolites (empty cavities) are extremely low in comparison to the binding towards the dye decorated binding sites. Thus, a simplification to a 1:1 binding model is applicable.

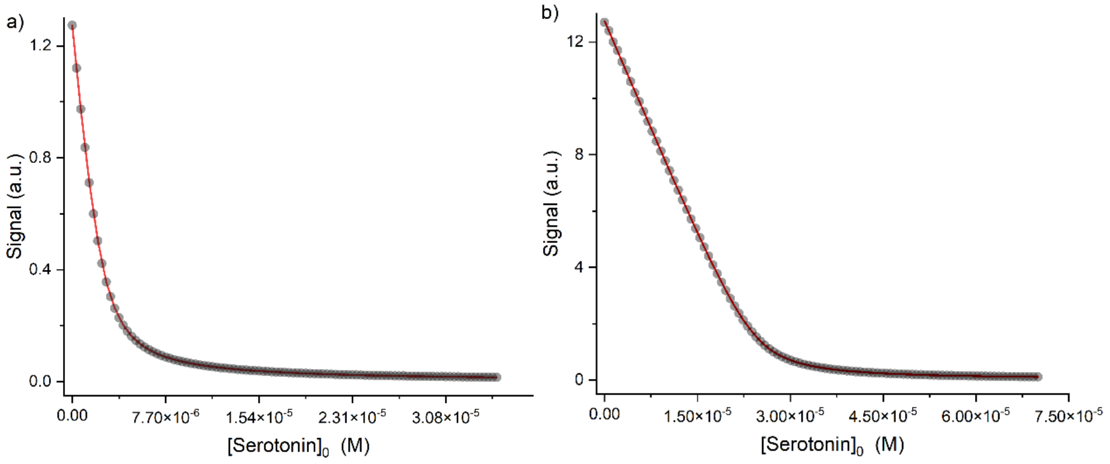


**Fig. S2.** Isotherm simulation of the binding of analytes (guests) towards ZARs using a competitive model with two binding sites ($C^e$ and $C^d$) shown as black dots and a 1:1 binding model (only $C^d$) shown as red line. (a) Low reporter dye loading (0.23 wt%), (b) high reporter dye loading (2.3 wt%).

Furthermore, the following equations demonstrate the validity of the 1:1 model by applying the given physicochemical conditions as approximations. The mathematical connection between the two possible equilibria is given by **Eq. S5**, demonstrating the competition of the cavities for the guest. The physicochemical conditions are applied throughout **Eq. S6** resulting in **Eq. S7** displaying the disconnection of the two competing equilibria.

$$[G]_0 = [G] + [C^e\mathrm{G}] + [C^d\mathrm{G}] \qquad \text{Eq. S5}$$

$$K_\mathrm{d}^\mathrm{e} \gg K_\mathrm{d}^\mathrm{d} \quad \rightarrow \quad [C^e\mathrm{G}] \ll [C^d\mathrm{G}] \qquad \text{Eq. S6}$$

$$[G]_0 = [G] + [C^d\mathrm{G}] \qquad \text{Eq. S7}$$

### 3.1. Data analysis and extraction of the dissociation constants $K_\mathrm{d}$

Binding affinities were determined by fluorescence and absorbance titration experiments by using equation 1, assuming that only the ZARs and the ZAR·NT complexes are emissive at the chosen emission wavelength:

$$\frac{F_{NT}}{F_0} = 1 + \frac{\Delta F[(c_{NT} + c_{ZAR} + K_d) - \sqrt{(c_{NT} + c_{ZAR} + K_d)^2 - 4 \cdot c_{NT} \cdot c_{ZAR}}]}{2 \cdot c_{ZAR}} \qquad \text{Eq. 1}$$

where $F_{NT}$ is the intensity at a given neurotransmitter (NT) concentration and $F_0$ is the emission intensity before neurotransmitter addition. $\Delta F$ is a measure of the relative emission increase or decrease caused by the neurotransmitter. For fully non-emissive ZAR·NT complexes, *i.e.*, when the neurotransmitter is an efficient quencher, $\Delta F$ reaches –1. The quantity $c_{NT}$ denotes the concentration of the neurotransmitter NT and $c_{ZAR}$ denotes the concentration of the "binding sites" in the ZAR. The values $K_d$ and $\Delta F$ result from the non-least square fit given the input $F_{NT}$, $F_0$, $c_{NT}$ and $c_{ZAR}$.

The concentration $c_{NT}$ of the neurotransmitter follows directly from the concentration of the stock solution and the added volume to the cuvette with the suspension of the ZAR. The concentration $c_{ZAR}$ of the single binding sites per ZAR suspension is *a priori* not known. However, $c_{ZAR}$ is directly and unambiguously obtained from the fitting of the binding isotherms for the case of strongly binding analytes, *e.g.*, serotonin. The so obtained concentrations of "binding sites" agreed well with the directly determined concentration of bound reporter dye in the ZARs. These was true for many different zeolite-dye loading ratios, verifying that the binding site are formed by the dye inside the zeolite cavities. The obtained value $c_{ZAR}$ was then used as a constant for the fitting of the binding isotherms of the weaker binding analytes. Notably, in all cases were fits observed with an adjusted R squared value > 0.98, which cooperates that all binding sites inside the zeolite crystal can be treated as independent and equal. In addition, it is reassuring that the value of the affinity constant $K_d$ is rather insensitive to the concentration $c_{ZAR}$.

## 4. Comparison between bioreceptors and artificial binders

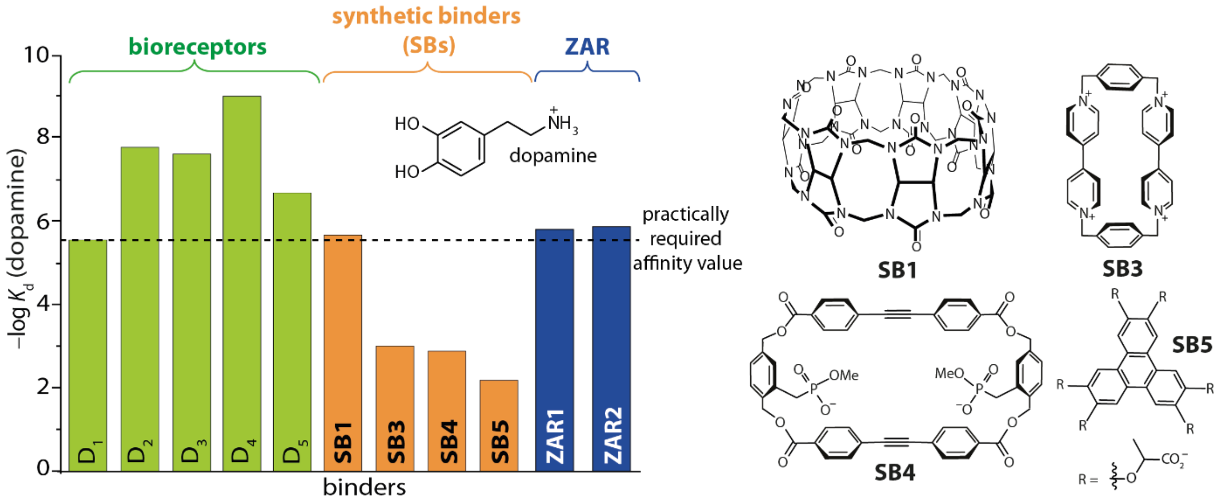


**Fig. S3.** Comparison of the binding affinity (depicted as -log $K_d$) for dopamine with natural bioreceptors (green), selected SBs (orange) and the herein introduced ZARs (blue). **SB1** shows the highest affinity amongst the shown SBs, however this macrocycle lacks selectivity and has no signal unit implemented. Detailed values are listed in **Table S3 - Table S5**.

**Table S3**. Serotonin receptors with their dissociation constants ($K_d$) for serotonin additionally to the used assay conditions and cell types/cell origins. The here shown list does not include all known serotonin receptors subtypes.

| Bioreceptor | $K_d$ (serotonin) nM | Assay conditions | Cell type / cell origin | Ref. |
|---|---|---|---|---|
| 5-$HT_{1B}$ | 2.5 | 50 mM Tris-HCl, pH 7.5 | rat striatum | 14 |
| 5-$HT_{2C}$ | 33 | 50 mM Tris-HCl, pH 7.5 | rat cortex | 14 |
| 5-$HT_3$ | 400 | 50 mM Tris-HCl, pH 7.5 | rat cortex | 15 |
| 5-$HT_4$ | 650<br>111 | 50 mM Tris-HCl, pH 7.4 | COS-7 cells<br>guinea pig striatum | 16<br>14 |

**Table S4.** Representative dopamine receptors and their dissociation constants for dopamine, serotonin, epinephrine, and norepinephrine additionally to the used assay conditions and cell types/cell origins. The here shown list does not include all known dopamine receptors.

| Bio-receptor | $K_d$ (dopamine) nM | $K_d$ (serotonin) nM | $K_d$ (epine-phrine) µM | $K_d$ (norepine-phrine) µM | Cell type / cell origin | Ref. |
|---|---|---|---|---|---|---|
| $D_1$ | 2340[a] | 9690[a] | 27000[a] | 32000[a] | COS-7 cells | 17,18 |
| $D_2$ | 16 [a] | n.d. | 5600[a] | 8300[a] | canine caudate nucleus membrane | 18,19 |
| $D_3$ | 25[b] | n.d. | n.d. | n.d. | kidney 293 cells | 20 |
| $D_4$ | 0.9[c] | n.d. | 13.8[c] | 33[c] | CHO cell | 21 |
| $D_5$ | 228[c] | 3000[c] | n.d. | n.d. | COS-7 cells | 17 |

n.d. = not determined; [a] 80 mM Tris-maleate, pH 7.4; [b] 50 mM Tris-HCL, pH 7.4, 100 mM NaCl; [c] 50 mM Tris-HCL, pH 7.4.

# 5. Photophysical properties

## 5.1. Excitation and emission spectra of ZARs

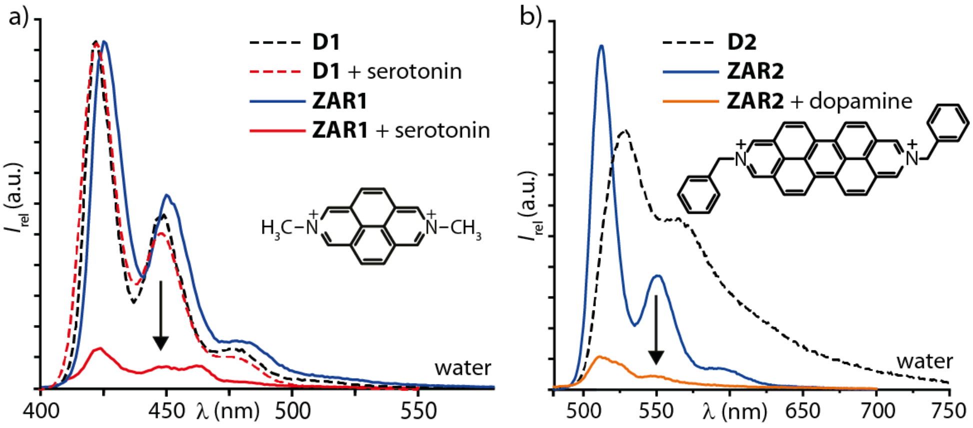


**Fig. S4.** (a) Normalized emission spectra of **D1** and its corresponding chemosensor **ZAR1** (250 µg mL$^{-1}$; 0.23 wt% dye loading) in the absence and presence of serotonin ($\lambda_{ex}$ = 371 nm) in water. Serotonin quenches the emission of **ZAR1,** but it does not interact with **D1** in aqueous media. (b) Normalized emission spectra of **D2** and its corresponding chemosensor **ZAR2** (250 µM mL$^{-1}$; 0.23 wt% dye loading) in the absence and presence of dopamine ($\lambda_{ex}$ = 450 nm) in water. Dopamine quenches the emission of **ZAR2**.

### 5.2. Fluorescence titration experiments and binding curves

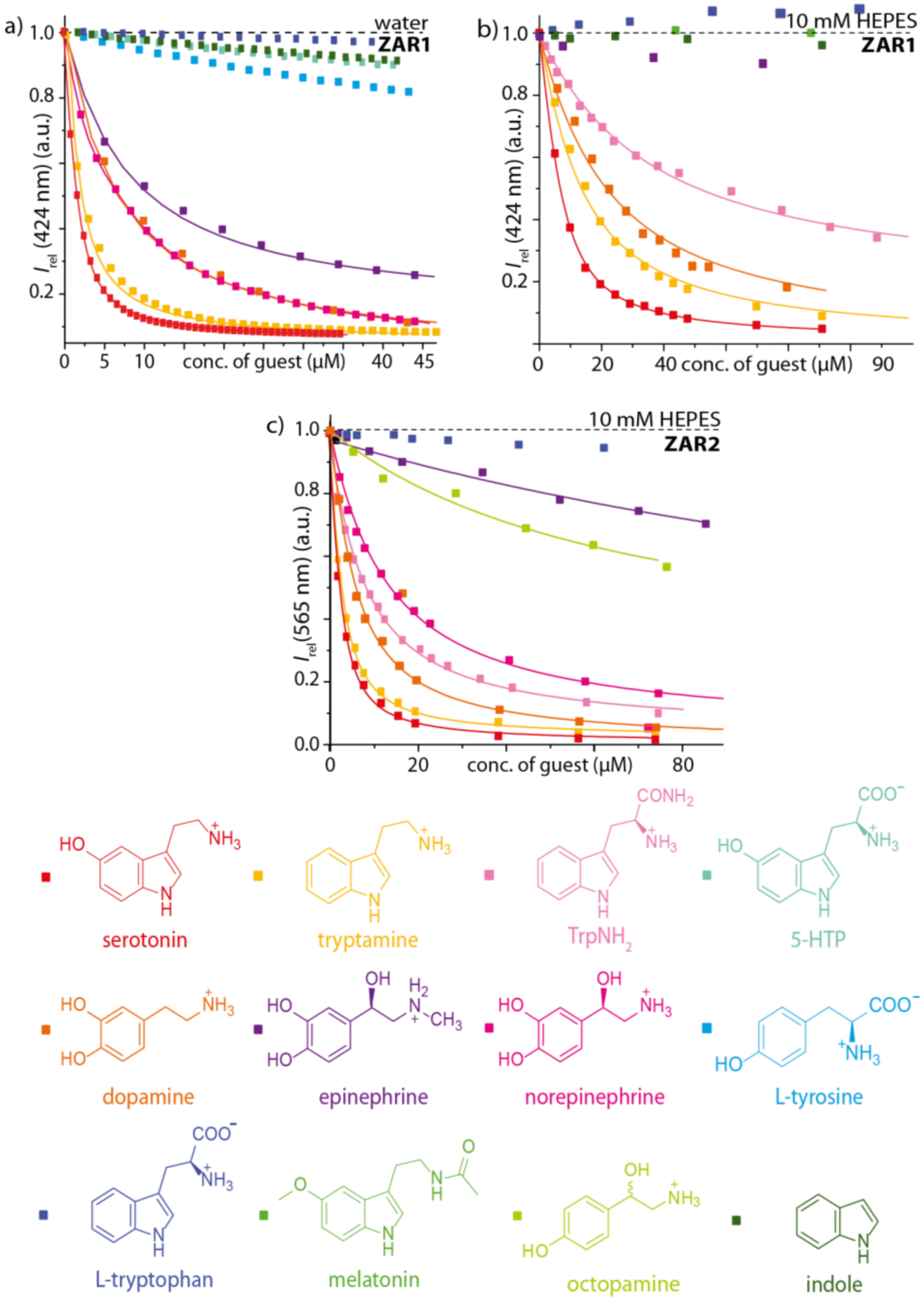


**Fig. S5.** Emission-based binding curves for several neurotransmitters and structural related molecules to **ZAR1** (250 µg mL$^{-1}$; 0.23 wt% dye loading) in (a) water ($\lambda_{ex}$ = 371 nm) and (b) 10 mM HEPES, pH 7.2, fitted with a 1:1 binding model. (c) Emission-based binding curves for several neurotransmitters and structural related molecules to **ZAR2** (250 µg mL$^{-1}$; 0.23 wt% dye loading) in 10 mM HEPES, pH 7.2, fitted with a 1:1 binding model ($\lambda_{ex}$ = 450 nm). For detailed binding values see **Table S5**.

**Table S5.** Detailed concentration information and averaged fitted single site dissociation constants ($K_d$) for the emission-based binding curves. Datasets were sorted alphabetically by analyte name. Used aqueous media were water, 10 mM HEPES, pH 7.2/7.3, 10 mM

phosphate buffer, pH 7.0, and 1X PBS, pH 7.0. Additionally, as biological relevant media neurobasal™ medium, HSA in 50 mM HEPES, pH 7.2, HS, and surine were used.

| ZAR | c(ZAR) (μg mL$^{-1}$) | Dye loading wt% (μM) | Guest | c(Guest) (μM) | Solvent | $\lambda_{ex}$ (nm) | $K_d$ (Average ± StDev) μM |
|---|---|---|---|---|---|---|---|
| ZAR1 | 250 | 0.23 (1.63) | dopamine | 0 - 39 | water | 371 | 5.1 ± 1.0 |
| | 250 | 0.23 (1.63) | | 0 - 153 | 10 mM HEPES | 371 | 11.3 ± 1.5 |
| | 250 | 0.23 (1.63) | | 0 - 130 | 10 mM HEPES + 10 mM NaCl | 371 | 13.5 ± 0.3 |
| | 250 | 0.23 (1.63) | | 0 - 177 | 10 mM HEPES + 50 mM NaCl | 371 | 24.0 ± 0.4 |
| | 250 | 0.23 (1.63) | | 0 - 107 | 10 mM phosphate buffer | 371 | 72.4 ± 2.8 |
| | 250 | 0.23 (1.63) | | 0 - 310 | 1X PBS | 371 | ≥ 250 |
| ZAR2 | 250 | 0.23 (0.81) | | 0 - 75 | water | 450 | 4.2 ± 0.8 |
| | 250 | 0.23 (0.81) | | 0 - 57 | 10 mM HEPES | 450 | 7.0 ± 0.5 |
| | 1500 | 0.23 (4.9) | | 0 - 120 | neurobasal™ medium | 300 | 48 ± 4.2 |
| | 780 | 0.23 (2.5) | | 0 - 14.5 | 250 μM HSA in 50 mM HEPES | 460 | 4.2 ± 0.7 |
| | 780 | 0.23 (2.5) | | 0 - 14.5 | HS : 50 mM HEPES (1:2) | 460 | 5.8 ± 0.5 |
| | 780 | 0.23 (2.5) | | 0 - 14.5 | surine | 460 | 7.8 ± 0.5 |
| ZAR $Y_{2.55}$2 | 250 | 0.23 (0.81) | | 0 - 100 | 10 mM HEPES (pH 7.3) | 450 | 9.5 ± 1.9 |
| ZAR3 | 250 | 0.23 (0.6) | | 0 - 85 | 10 mM HEPES | 445 | 9.7 ± 2.0 |
| ZAR $Y_{15}$1 | 250 | 0.23 (1.63) | dopamine | 0 - 55 | water | 371 | 3.0 ± 0.2 |
| ZAR $Y_{15}$1 | 250 | 0.23 (1.63) | | 0 - 55 | water + 500 μM ascorbic acid | 371 | 2.9 ± 0.2 |
| ZAR1 | 250 | 0.23 (1.63) | epinephrine | 0 - 140 | water | 371 | 11.0 ± 0.8 |

| | | | | | | | |
|---|---|---|---|---|---|---|---|
| | 250 | 0.23 (1.63) | | 0 - 260 | 10 mM HEPES | 371 | 57.6 ± 3.3 |
| | 250 | 0.23 (1.63) | | 0 - 170 | 10 mM phosphate buffer | 371 | 357 ± 30 |
| | 250 | 0.23 (1.63) | | 0 - 330 | 1X PBS | 371 | ≥ 450 |
| **ZAR2** | 250 | 0.23 (0.81) | | 0 - 150 | 10 mM HEPES | 442 | 240 ± 48 |
| **ZAR $Y_{2.55}$1** | 250 | 0.23 (1.63) | | 0 - 100 | 10 mM HEPES | 395 | 39 ± 8 |
| **ZAR $Y_{2.55}$2** | 250 | 0.23 (0.81) | histamine | 0 - 275 | 10 mM HEPES | 475 | 56 ± 11 |
| **ZAR3** | 250 | 0.23 (0.6) | | 0 - 85 | 10 mM HEPES | 445 | 360 ± 72 |
| **ZAR1** | 250 | 0.23 (1.63) | 5-HTP | 0 - 100 | water | 371 | ≥ 130 |
| **ZAR1** | 250 | 0.23 (1.63) | indole | 0 - 118 | water | 371 | ≥ 130 |
| **ZAR $Y_{15}$1** | 250 | 0.23 (1.63) | | 0 - 60 | water | 371 | 5.0 ± 0.3 |
| **ZAR1** | 250 | 0.23 (1.63) | norepine-phrine | 0 - 65 | water | 371 | 5.1 ± 0.3 |
| | 250 | 0.23 (1.63) | | 0 - 100 | 10 mM HEPES | 371 | 4.8 ± 0.4 |
| | 250 | 0.23 (1.63) | | 0 - 100 | 10 mM phosphate buffer | 371 | 12.8 ± 0.4 |
| | 250 | 0.23 (1.63) | | 0 - 150 | 1X PBS | 371 | 100 ± 20 |
| **ZAR2** | 250 | 0.23 (1.63) | | 0 - 90 | 10 mM HEPES | 442 | 13 ± 2.6 |
| **ZAR $Y_{2.55}$1** | 250 | 0.23 (1.63) | | 0 - 150 | 10 mM HEPES | 395 | 26 ± 5.2 |
| **ZAR1** | 250 | 0.23 (1.63) | octo-pamine | 0 - 90 | 10 mM HEPES | 395 | 40 ± 8.0 |
| **ZAR2** | 250 | 0.23 (0.81) | octo-pamine | 0 - 80 | 10 mM HEPES | 442 | 68 ± 13 |
| **ZAR3** | 250 | 0.23 (0.6) | phenethyl amine | 0 - 85 | 10 mM HEPES (pH 7.3) | 445 | 190 ± 38 |

| | | | | | | | |
|---|---|---|---|---|---|---|---|
| ZAR1 | 250 | 0.23 (1.63) | serotonin | 0 - 15.3 | water | 371 | 0.3 ± 0.1 |
| | 250 | 2.3 (16.3) | | 0 - 34 | water | 371 | 0.2 ± 0.1 |
| | 250 | 0.23 (1.63) | | 0 - 9 | water + 50 mM Trp | 400 | 14 ± 2.8 |
| | 250 | 0.23 (1.63) | | 0 - 16.5 | water + 5 µM cadaverine | 371 | 0.2 ± 0.1 |
| | 250 | 0.23 (1.63) | | 0 - 16.5 | water + 10 µM cadaverine | 371 | 0.3 ± 0.1 |
| | 250 | 0.23 (1.63) | | 0 - 16.5 | water + 50 µM cadaverine | 371 | 1.0 ± 0.1 |
| | 250 | 0.23 (1.63) | | 0 - 105 | 10 mM HEPES | 371 | 1.2 ± 0.2 |
| | 250 | 0.23 (1.63) | | 0 - 57 | 10 mM phosphate buffer | 371 | 1.8 ± 0.2 |
| | 250 | 0.23 (1.63) | | 0 - 107 | 1X PBS | 371 | 14.6 ± 0.4 |
| | 250 | 0.23 (1.63) | | 0 - 90 | ethanol | 396 | ~ 25 |
| | 250 | 0.23 (1.63) | | 0 - 90 | ethanol + 0.2 mM acetic acid | 396 | ~ 25 |
| ZAR1 -DXP | 250 | 0.23 (1.63) | | 0 - 30 | 10 mM HEPES (pH 7.3) | 360 | 1.4 ± 0.4 |
| | 25 | 0.23 (1.63) | | 0 - 0.8 | 10 mM HEPES (pH 7.3) | 360 | n.d. |
| ZAR2 | 250 | 0.23 (0.81) | | 0 - 15 | water | 450 | 0.48 ± 0.03 |
| | 250 | 0.23 (0.81) | | 0 - 15 | 10 mM HEPES | 450 | 0.71 ± 0.08 |
| | 1500 | 0.23 (4.9) | | 0 - 53 | neurobasal™ medium | 300 | 1.0 ± 0.1 |
| | 780 | 0.23 (2.5) | | 0 - 14.5 | 250 µM HSA in 50 mM HEPES | 460 | 1.6 ± 0.4 |
| | 780 | 0.23 (2.5) | | 0 - 14.5 | HS : 50 mM HEPES (1:2) | 460 | 3.7 ± 0.9 |
| | 780 | 0.23 (2.5) | | 0 - 14.5 | surine | 460 | 2.6 ± 0.2 |

| | | | | | | | |
|---|---|---|---|---|---|---|---|
| **ZAR $Y_{2.55}$1** | 250 | 0.23 (1.63) | serotonin | 0 - 7.5 | 10 mM HEPES | 395 | 0.63 ± 0.13 |
| **ZAR3** | 250 | 0.23 (0.6) | | 0 - 85 | 10 mM HEPES | 445 | 2.7 ± 0.5 |
| **ZAR $Y_{15}$1** | 250 | 0.23 (1.63) | | 0 - 55 | water | 371 | 0.5 ± 0.1 |
| | 250 | 0.23 (1.63) | | 0 - 55 | water + 500 µM ascorbic acid | 371 | 0.9 ± 0.1 |
| **ZAR1** | 250 | 0.23 (1.63) | $TrpNH_2$ | 0 - 123 | 10 mM HEPES (pH 7.3) | 395 | 32 ± 6.4 |
| **ZAR2** | 250 | 0.23 (0.81) | | 0 - 90 | 10 mM HEPES (pH 7.3) | 442 | 6.7 ± 1.3 |
| **ZAR1** | 250 | 0.23 (1.63) | tryptamine | 0 - 35 | water | 395 | 1.05 ± 0.05 |
| | 250 | 0.23 (1.63) | | 0 - 125 | 10 mM HEPES (pH 7.3) | 395 | 1.0 ± 0.2 |
| **ZAR2** | 250 | 0.23 (0.81) | | 0 - 76 | 10 mM HEPES (pH 7.3) | 442 | 1.6 ± 0.3 |
| **ZAR1** | 250 | 0.23 (1.63) | L-Trp | 0 - 140 | 10 mM HEPES | 371 | ≥ 600 |
| **ZAR $Y_{15}$1** | 250 | 0.23 (1.63) | | 0 - 55 | water | 371 | 5.4 ± 0.8 |
| **ZAR1** | 250 | 0.23 (1.63) | tyramine | 0 - 70 | water | 371 | 6.2 ± 0.5 |
| | 250 | 0.23 (1.63) | | 0 - 120 | 10 mM HEPES | 395 | 27 ± 5.4 |
| **ZAR2** | 250 | 0.23 (0.81) | | 0 - 95 | 10 mM HEPES | 442 | 13 ± 2.6 |

### 5.3. Ratiometric sensing experiments

**DXP** (100 µg) was mixed with commercial zeolite $L_{3.0}$ (100 mg) in a glass ampoule.[22] The amount of **DXP** loading was calculated considering that one **DXP** occupies 3 unit cells and a loading of 20% corresponds to the highest theoretical loading possible. A loading of 1% was aimed for. The ampoule was dehydrated at about $4.0 \cdot 10^{-6}$ mbar for 12 h. The ampoule was heated to 300°C for 12 h in the rotating oven. Afterwards, the zeolite $L_{3.0}$ crystals were

washed with n-butanol until the supernatant showed no absorbance anymore and dried in vacuum.

To a suspension of **DXP-loaded zeolite L** in water (20 mg $mL^{-1}$), **D1** was added to achieve a concentration of 1 mM. The suspension was shacked, centrifuged and the solid was treated 4x with a washing-centrifugation-decanting sequence and dried in vacuum.

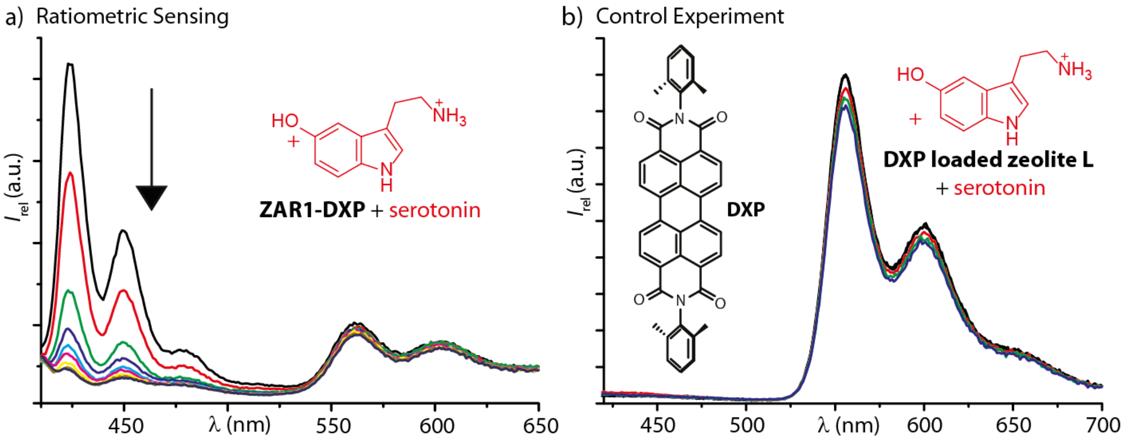


**Fig. S6.** Ratiometric sensing with **ZAR1** that contains a co-encapsulated, non-analyte responsive reference dye **DXP** (= **ZAR1-DXP**). (a) The detection of serotonin (c = 0 - 40 μM) with **ZAR1-DXP** (250 μg $mL^{-1}$; 0.23 wt% dye loading). The neurotransmitter binding quenches the emission of the zeolite-bound reporter dye **D1** (400 - 525 nm) but does not affect the emission of the zeolite-bound **DXP** (540 - 650 nm) in 10 mM HEPES, pH 7.3. (b) Control experiment in the absence of reporter dye **D1** in 10 mM HEPES, pH 7.3 ($\lambda_{ex}$ = 360 nm).

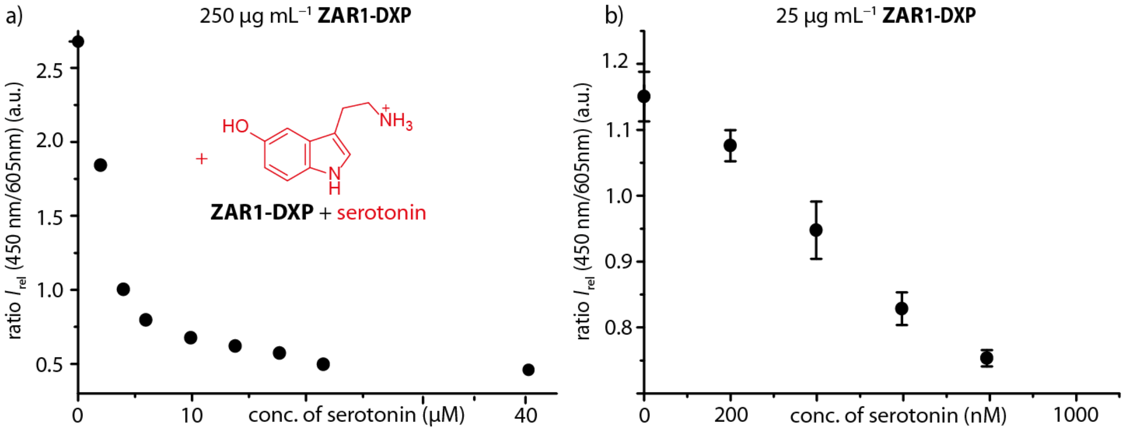


**Fig. S7.** Intensity ratio of the emission intensity at 450 nm (corresponding to **D1**) and the emission intensity at 605 nm (corresponding to **DXP**) while titrating serotonin into (a) 250 μg $mL^{-1}$ **ZAR1-DXP** (0.23 wt% dye loading) and (b) 25 μg $mL^{-1}$ **ZAR1-DXP** (0.23 wt% dye loading) in 10 mM HEPES, pH 7.3 ($\lambda_{ex}$ = 360 nm).

### 5.4. Absorbance-based measurements

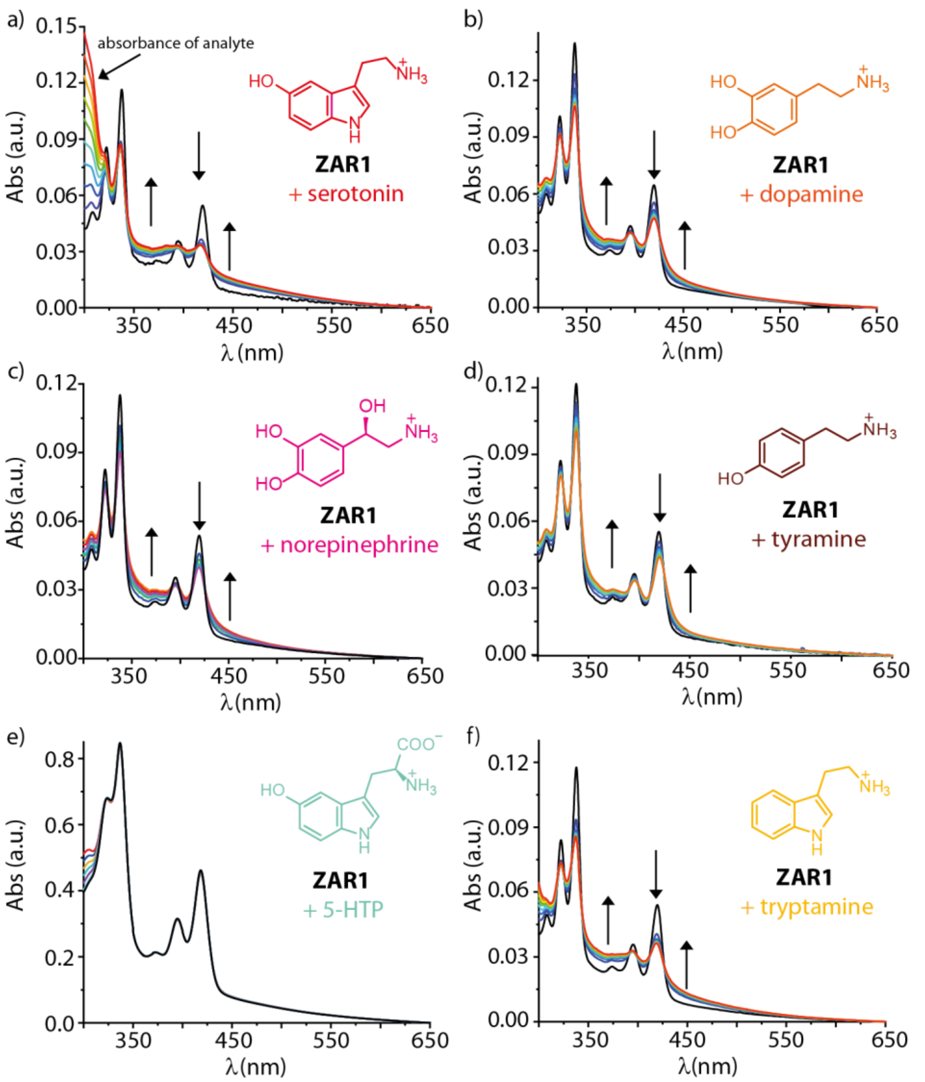


**Fig. S8.** Absorbance-based titration of (a) serotonin (c = 0 - 21 µM), (b) dopamine (c = 0 - 48 µM), (c) norepinephrine (c = 0 - 21 µM), (d) tyramine (c = 0 - 22 µM), (e) 5-hydroxytryptophan (5-HTP, c = 0 - 34 µM) and (f) tryptamine (c = 0 - 21 µM) to an aqueous dispersion of **ZAR1** (250 µg mL$^{-1}$; 2.3 wt% dye loading). The addition of all analytes except for 5-HTP strongly alters the absorbance spectrum of **ZAR1**, *e.g.*, charge transfer bands emerge in the region of 430 - 550 nm. The zwitterionic 5-HTP does only very weakly bind to ZARs and thus does not cause a change of the absorbance of the ZARs.

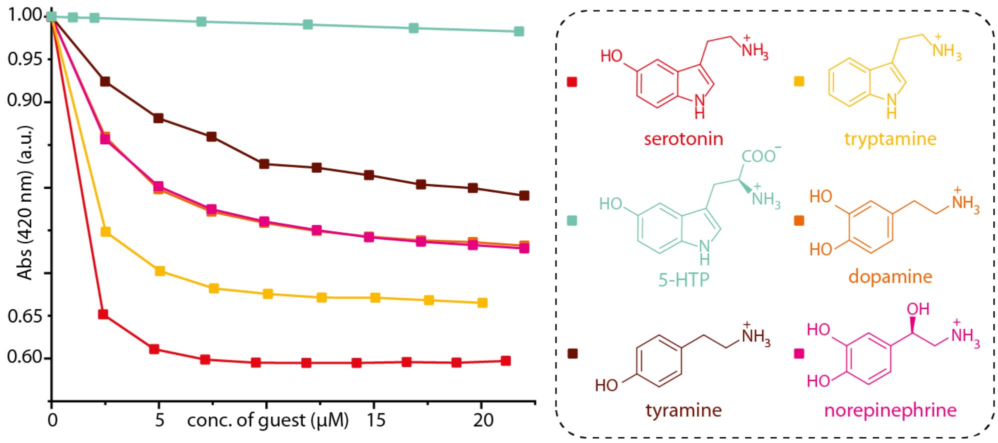


**Fig. S9.** Summary of the absorbance changes at 420 nm under the addition of the here depicted analytes to **ZAR1** (250 µg mL$^{-1}$; 2.3 wt% dye loading). All analytes can be distinguished based on absorbance spectral data except for dopamine and norepinephrine, which differ in one hydroxy group.

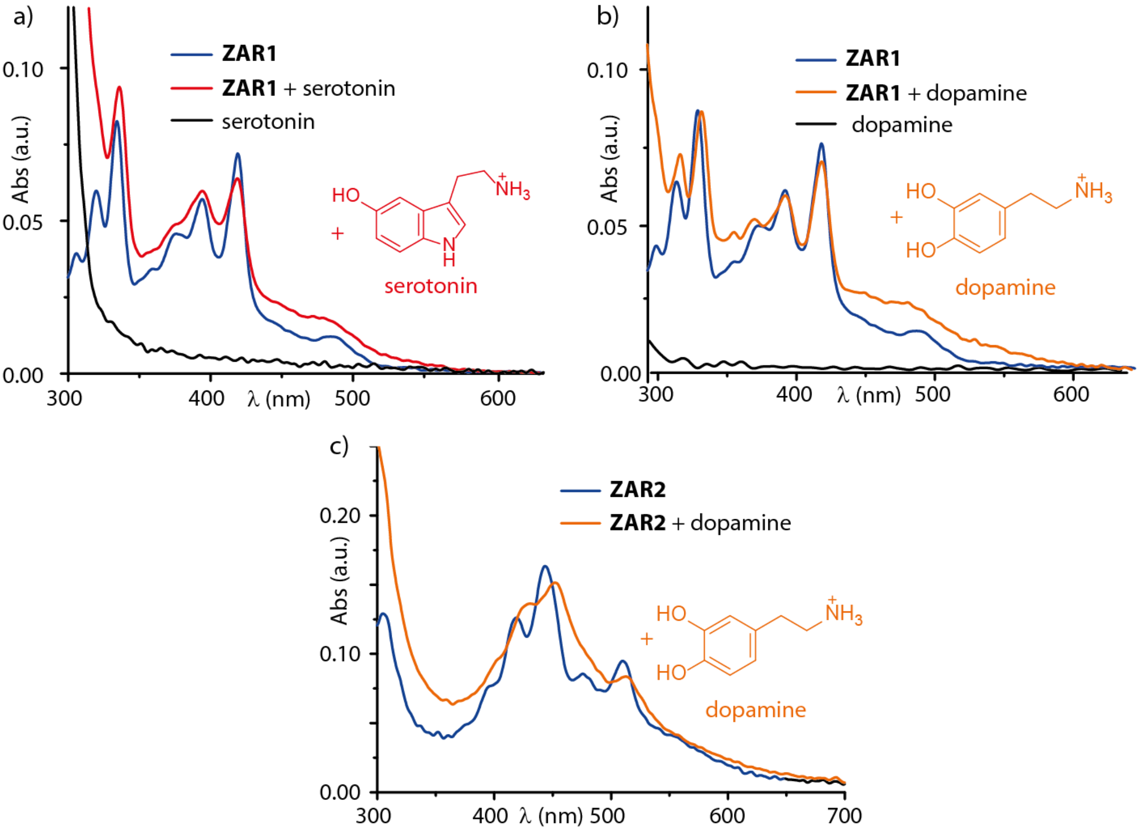


**Fig. S10.** Solid state absorbance spectra of **ZAR1** (250 µg mL$^{-1}$; 2.3 wt% dye loading) (a) in the absence and presence of serotonin and (b) in the absence and presence of dopamine. For comparison, the solid state absorbance spectrum of the analytes are depicted. (c) Solid state absorbance spectra of **ZAR2** (250 µg mL$^{-1}$; 2.3 wt% dye loading) in the absence and presence of dopamine.

### 5.5. Two-photon microcopy

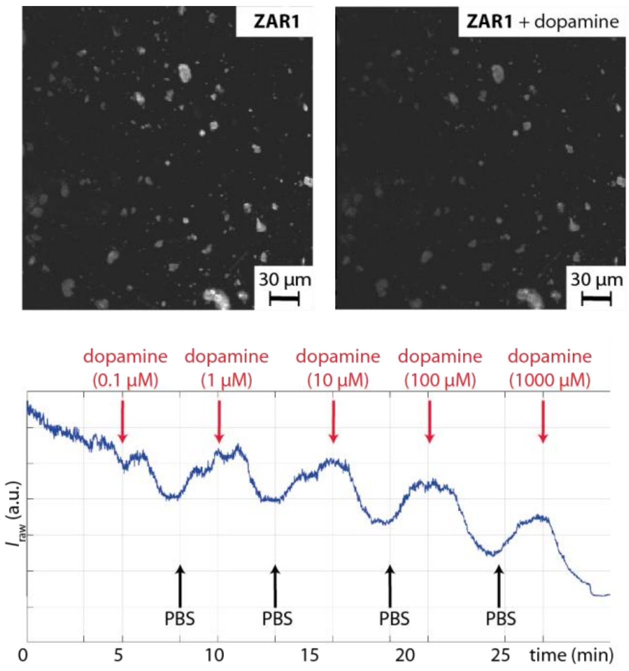


**Fig. S11.** Two-photon microscopy experiments with **ZAR1** particles that were entrapped by an *in situ* formed fibroin gel deposited on a microscopy coverslip. In contrast to solution experiments where **ZAR1** particles are monodispersed, also clusters of ZAR particles are observed on the surface. The coverslip was mounted on the microscopy stage and then exposed for 15 min to PBS as a pre-equilibration step, followed by flushing the system with a solution of dopamine (100 µM) in PBS.

## 6. Selectivity

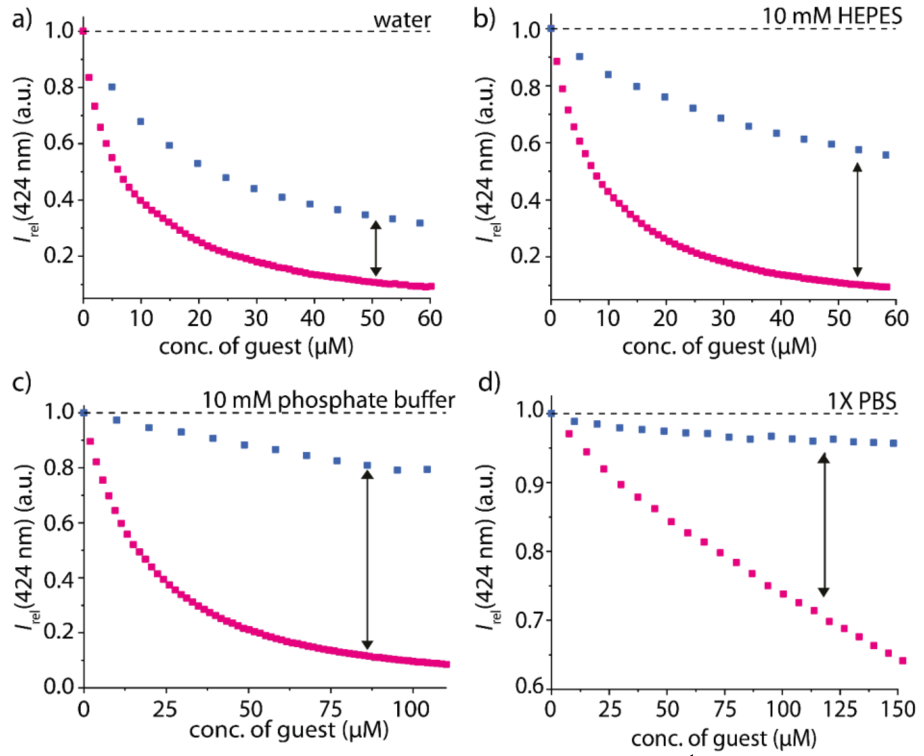


**Fig. S12.** Binding curves for the titration of **ZAR1** (250 µg mL$^{-1}$; 0.23 wt% dye loading) with epinephrine (blue) and norepinephrine (pink) in water, 10 mM HEPES buffer (pH 7.2, total conc. of cations $c_{cat,\ tot}$ ~ 10 mM), 10 mM phosphate buffer (pH 7.0, $c_{cat,\ tot}$ ~ 17.5 mM) and saline phosphate buffer (1X PBS, pH 7.0, $c_{cat,\ tot}$ ~ 202 mM). The stronger binder norepinephrine is much less affected by increasing salt concentrations compared to the weaker binder epinephrine.

**Table S6.** Representative artificial binders for epinephrine and norepinephrine are listed with their systematic host name, their dissociation constants for epinephrine and norepinephrine additionally to their selectivity for norepinephrine over epinephrine. If not stated otherwise, the given values were determined in water.

| | **Systematic host name** | **$K_d$ (epinephrine) µM** | **$K_d$ (norepinephrine) µM** | **Selectivity** | **Ref.** |
|---|---|---|---|---|---|
| **SB3** | cyclobis(paraquat-1,4-phenylene)$^{4+}$ | 1667 | 676 | 2.5 | 23 |
| **SB4** | 2,19,28,45-tetraoxa-3,18,29,44-tetraoxo-10,36-diyne-22,25,48,51-tetrakis(methoxy-phosphoryl-methyl)-[3.3.2] paracyclophane$^{2-}$ | 813[a] | 800[a] | 1.0 | 24 |
| **SB5** | triphenylene-2,3,6,7,10,11-hexaylhexakis(oxy)hexa-propionate | 4902 | 5376 | 1.1 | 25 |
| **$\alpha_{2A}$** | / | 0.4[b] | 1.7[b] | 4.6 | 26 |
| **$\beta_2$** | / | 0.7 | / | / | 27 |

n.d. = not determined; [a] $D_2O$; [b] 25 mM Tris-HCl, pH 7.4.

**Table S7.** Representative artificial binders for serotonin and dopamine are listed with their systematic host name, their dissociation constants for serotonin and dopamine, additionally to

the used conditions and their selectivity for serotonin over dopamine. If not stated otherwise, the given values were determined in water.

| | Systematic host name | $K_d$ (serotonin) µM | $K_d$ (dopamine) µM | Selectivity | Ref. |
|---|---|---|---|---|---|
| **SB1** | cucurbit[7]uril | 14.6 | 2.2 | 6.6 | 28 |
| **SB2** | *β*-cyclodextrin | 18868 | n.d. | / | 29 |
| **SB3** | cyclobis(paraquat-1,4-phenylene)$^{4+}$ | 645 | 935 | 1.4 | 23 |
| **SB4** | 2,19,28,45-Tetraoxa-3,18,29,44-tetraoxo-10,36-diyne-22,25,48,51-tetrakis (methoxyphosphoryl-methyl)-[3.3.2.$_2$]paracyclophane$^{2-}$ | 617[a] | 1150[a] | 1.9 | 24 |
| **SB5** | triphenylene-2,3,6,7,10,11-hexaylhexakis(oxy)hexa-propionate | 4975[b] | 5155[b] | 1.0 | 25 |
| **SB6** | cucurbit[8]uril decorated with **D1** | 200 | n.d. | / | 30 |

n.d. = not determined; [a] $D_2O$; [b] 100 mM $Na_2HPO_4$, pH 7.1.

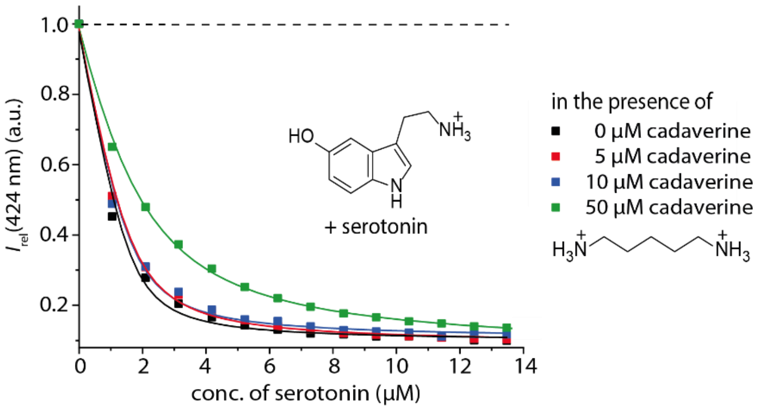


**Fig. S13.** Binding curves for the titration of **ZAR1** (250 µg mL$^{-1}$; 0.23 wt% dye loading) with serotonin in water in the absence and presence of cadaverine. The excitation wavelength $\lambda_{ex}$ = 371 nm was used. The solid lines represent the least-square fit to a single-site 1:1 binding model.

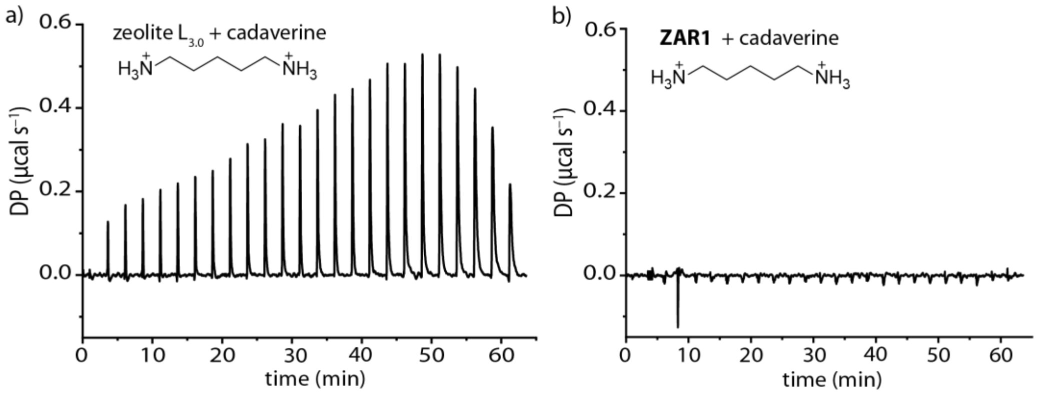

**Fig. S14.** Raw heat ITC thermogram for the titration of (a) zeolite $L_{3.0}$ nanoparticles (250 µg mL$^{-1}$) and (b) **ZAR1** nanoparticles (250 µg mL$^{-1}$; 2.3 wt% dye loading) with the doubly charged biogenic amine cadaverine. Serotonin binding to **ZAR1** is not strongly affected by the biogenic diamine cadaverine, which naturally occurs in a low micromolar concentration range in biofluids. For comparison, **SB1** is dysfunctional for neurotransmitter capture in the presence of cadaverine and other biogenic amines.

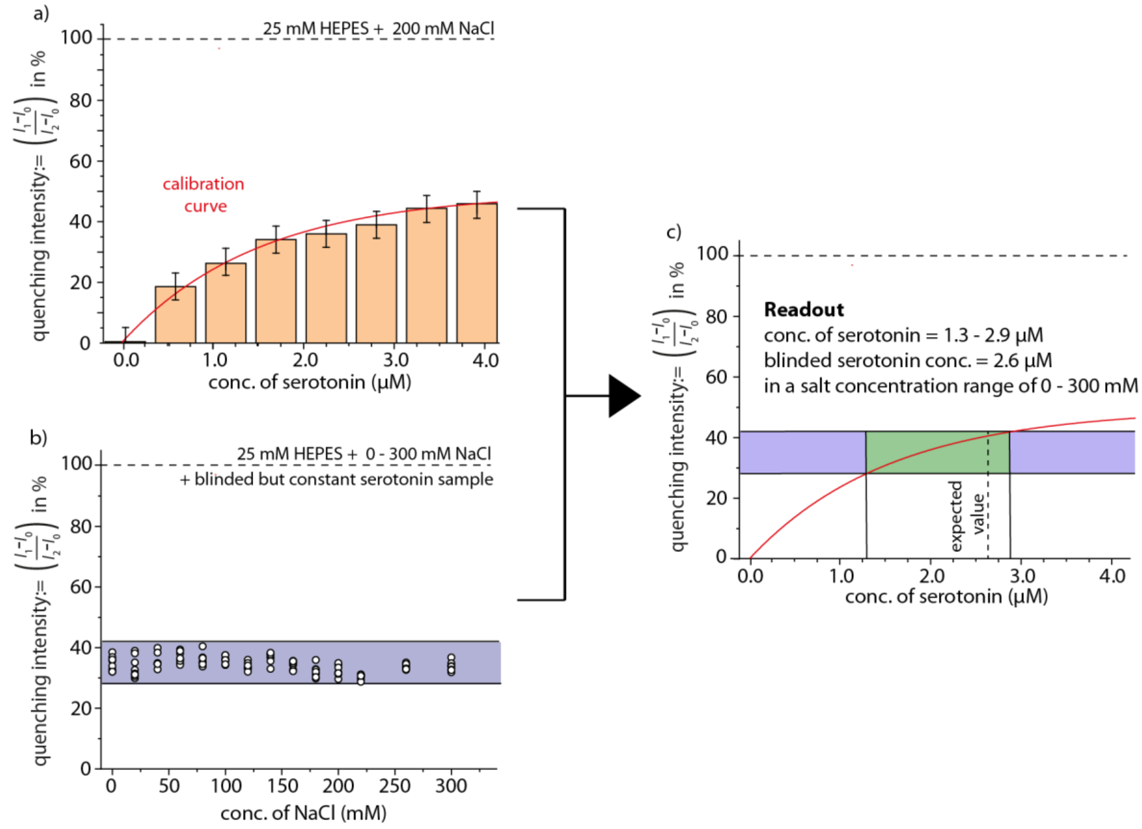


**Fig. S15.** Experiments to emulate a potential salt influence ("matrix effect") on serotonin detection by **ZAR1.** (a) To a dispersion of **ZAR1** (250 µg mL$^{-1}$; 2.3 wt% dye loading) in 25 mM HEPES, pH 7.2, and 200 mM NaCl were added several aliquots of serotonin and the quenching intensity was used to obtain a calibration curve. Given errors are standard deviations. (b) The quenching intensity of an unknown serotonin solution was determined by a ZAR-based assay in the presence of different amounts of salts (n = 8 for each salt concentration). (c) Combining the information obtained in a) and b), the ZAR-assay based serotonin concentration was determined by comparison of the emission quenching ratio to that of an independently obtained calibration curve. The green box depicts the potential influence of an unknown salt content present in the medium, herein shown by a range of 0 to 300 mM NaCl, on the calibration curve-based determination of the serotonin concentration. The 'real' serotonin concentrations in the spiked samples is depicted as dashed line. An excitation wavelength of $\lambda_{ex}$ = 395 nm was used and the emission was recorded at $\lambda_{em}$ = 426 nm.

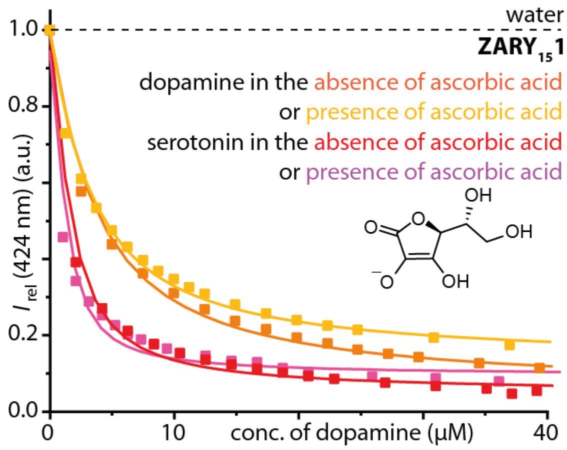


**Fig. S16.** Binding curves for the titration of **ZARY$_{15}$1** (250 µg mL$^{-1}$; 0.23 wt% dye loading) with serotonin and dopamine, each in water (serotonin - red; dopamine - orange) and in water to which were added 500 µM ascorbic acid (serotonin - violet, dopamine - yellow). The excitation wavelength $\lambda_{ex}$ = 371 nm was used. The solid lines represent the least-square fit to a single-site 1:1 binding model. The binding strength of serotonin and dopamine is almost not affected from the presence of ascorbic acid. The same results were found for **ZAR1** (250 µg mL$^{-1}$; 0.23 wt% dye loading) in the presence of 500 µM ascorbic acid.

## 7. Fluorescence titration experiments in biological relevant media

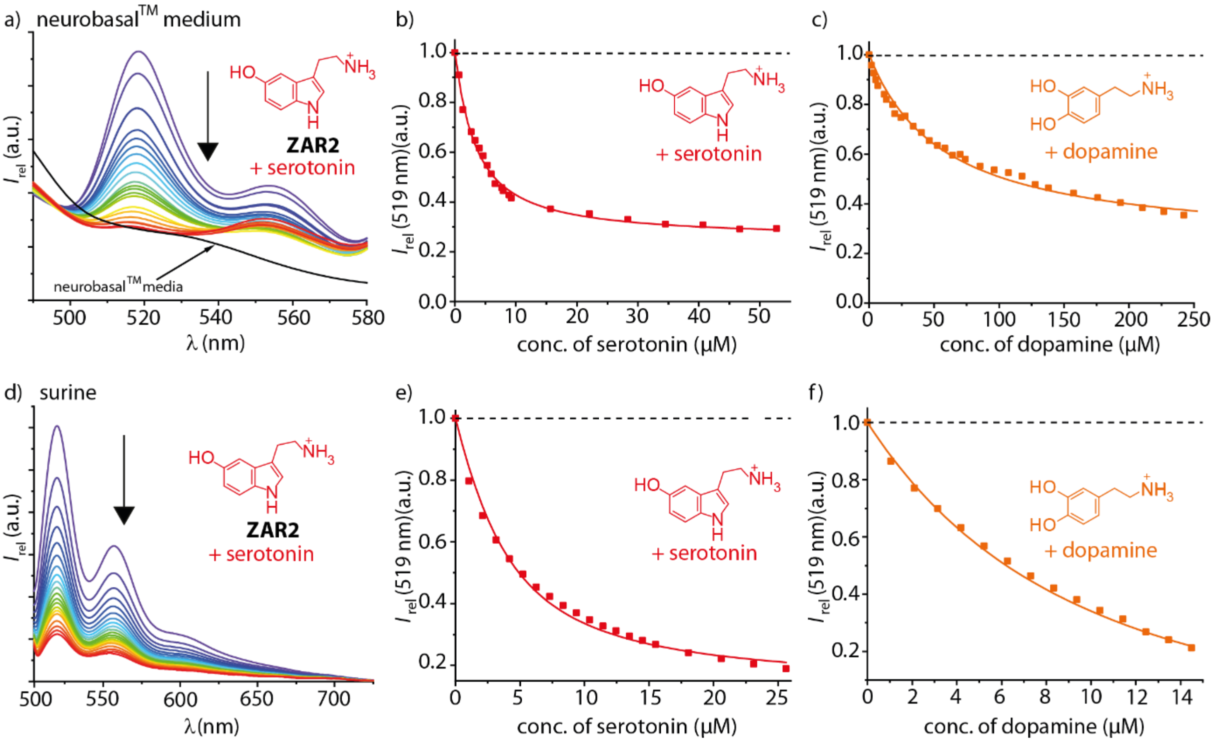


**Fig. S17.** (a) Emission spectra for the titration of **ZAR2** (1.5 mg mL$^{-1}$; 0.23 wt% dye loading) with serotonin (c = 0 - 53 µM) in neurobasal$^{TM}$ medium. The excitation wavelength $\lambda_{ex}$ = 300 nm was used. The corresponding binding curves with (b) serotonin (c = 0 - 53 µM, red) and (c) dopamine (c = 0 - 250 µM, orange) were monitored at $\lambda_{em}$ = 519 nm. Fits were

con-ducted with a 1:1 binding site model, yielding in a single site dissociation constant $K_d$ = 1.0 µM for serotonin and a single site dissociation constant $K_d$ = 48 µM for dopamine. (d) Emission spectra for the titration of **ZAR2** (780 µg mL$^{-1}$; 0.23 wt% dye) with serotonin (c = 0 - 52.0 µM) in surine diluted 1:1 with 50 mM HEPES buffer, pH 7.2. The excitation wavelength $\lambda_{ex}$ = 460 nm was used. The corresponding binding curves with (e) serotonin (c = 0 - 52.0 µM, red) and (f) dopamine (c = 0 - 250 µM, orange) were monitored at $\lambda_{em}$ = 519 nm. Fits were conducted with a 1:1 binding site model, yielding in a single site dissociation constant $K_d$ = 2.6 µM for serotonin and a single site dissociation constant $K_d$ = 7.8 µM for dopamine.

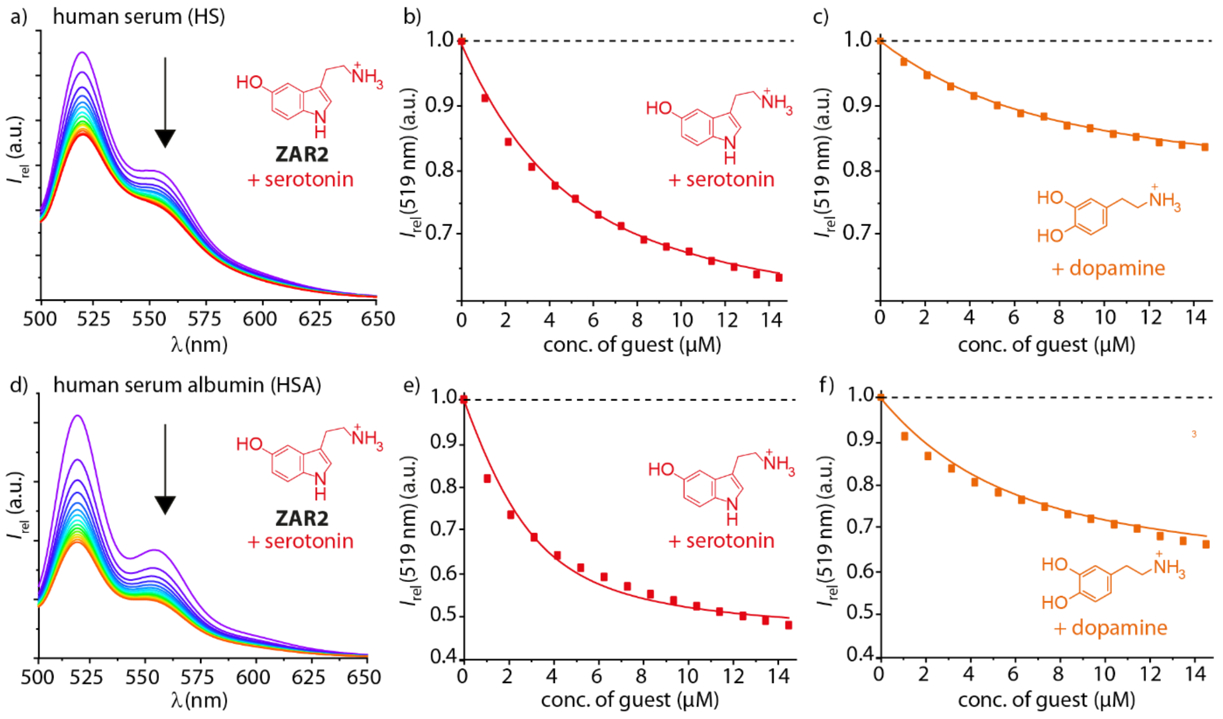


**Fig. S18.** (a) Emission spectra for the titration of **ZAR2** (780 µg mL$^{-1}$; 0.23 wt% dye loading) with serotonin (c = 0 - 14.5 µM) in human serum (HS) (HS/50mM HEPES, pH 7.2, 1:2 v/v) resulting in a quenched ZAR emission. Data was corrected for autofluorescence of HS. The excitation wavelength $\lambda_{ex}$ = 460 nm was used. The corresponding binding curves with (b) serotonin (c = 0 - 14.5 µM, red) and (c) dopamine (c = 0 - 14.5 µM, orange) were monitored at $\lambda_{em}$ = 519 nm. Fits were conducted with a 1:1 binding site model, yielding in a single site dissociation constant $K_d$ = 3.3 µM for serotonin and a single site dissociation constant $K_d$ = 5.8 µM for dopamine. (d) Emission spectra for the titration of **ZAR2** (780 µg mL$^{-1}$; 0.23 wt% dye loading) with serotonin (c = 0 - 14.5 µM) in human serum albumin (HSA, fatty acid free, c(HSA) = 250 µM) in 50 mM HEPES buffer, pH 7.2. Data was corrected for autofluorescence of HSA. The excitation wavelength $\lambda_{ex}$ = 460 nm was used. The corresponding binding curve with (e) serotonin (c = 0 - 14.5 µM, red) and (f) dopamine (c = 0 - 14.5 µM, orange) were monitored at $\lambda_{em}$ = 519 nm. Fits were conducted with a 1:1 binding site model, yielding in a single site dissociation constant $K_d$ = 1.3 µM for serotonin and a single site dissociation constant $K_d$ = 4.2 µM for dopamine.

## 8. Assays in synthetic urine (surine) and human urine

To simulate levels of serotonin with different pathological outcome, artificial urine (surine) was spiked with several serotonin concentrations. To mimic low neurotransmitter concentrations, three samples were spiked with <0.5 µM serotonin, for normal neurotransmitter levels, three samples were spiked with 1.0 - 2.5 µM serotonin and for elevated neurotransmitter levels, three samples were spiked with ≤10 µM serotonin. Additionally, 0 or 3 µM dopamine were added to the spiked samples to identify the influence of dopamine presence on the ZARs. The intensity of **ZAR1** (320 µg mL$^{-1}$, 2.3 wt% dye) dispersed in 50 mM HEPES, pH 7.2, was monitored before and after addition of the sample ($\lambda_{ex}$ = 395 nm; $\lambda_{ex}$ = 426 nm) and then the percentage intensity quenching was calculated. A **ZAR1**-based assay (250 µg mL$^{-1}$; 0.23 wt% dye loading) can distinguish low (0.3 µM – blue; 1.2 µM – green) from normal (2.1 µM – red) serotonin levels in surine (n = 16 for each serotonin concentration).

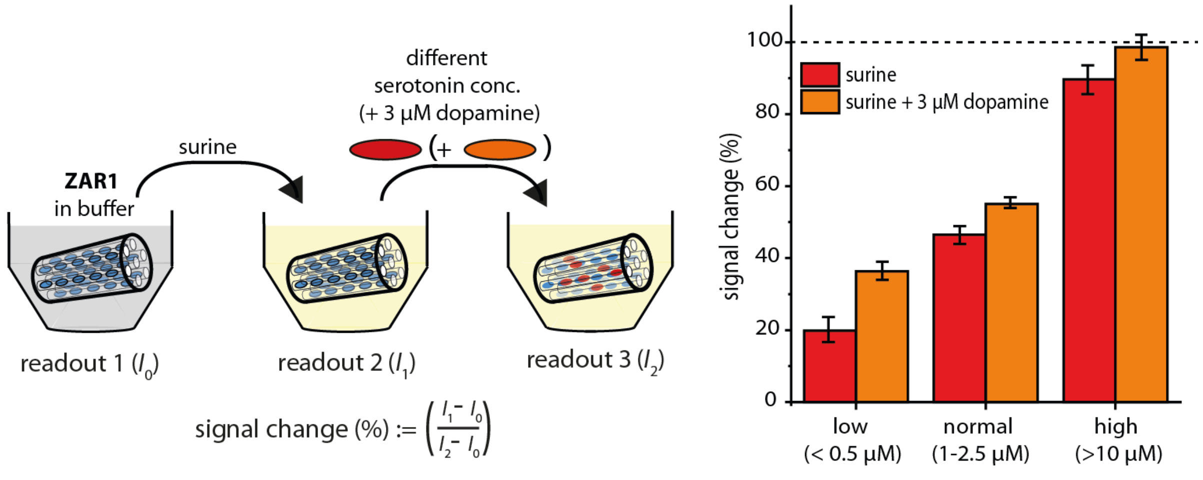


**Fig. S19.** Schematic representation of a ZAR-based assay for distinguishing abnormally low neurotransmitter levels from that of the normal range in biofluids, here shown for surine: To a dispersion of **ZAR1** in 50 mM HEPES (pH 7.2, readout 1) was added surine (readout 2) and then several concentrations of serotonin and mixtures of serotonin with 3 µM dopamine were added (readout 3). The % intensity quenching was calculated out of the three readouts using the displayed equation. Bar graph for the percentage of emission quenching of **ZAR1** (250 µg mL$^{-1}$; 2.3 wt% dye loading) of different serotonin/dopamine spiked surine samples (n = 3 for each low, normal, and high serotonin levels) in analogy to the depicted scheme. Given errors are standard deviations. The excitation wavelength $\lambda_{ex}$ = 395 nm and the emission wavelength $\lambda_{em}$ = 426 nm were used.

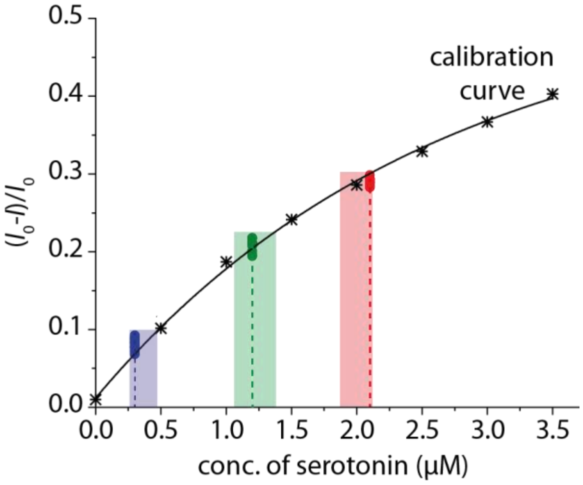


**Fig. S20.** A **ZAR1**-based assay (250 µg $mL^{-1}$; 0.23 wt% dye loading) can distinguish low (0.3 µM – blue; 1.2 µM – green) from normal (2.1 µM – red) serotonin levels in surine (n = 16 for each serotonin concentration). The 'real' serotonin concentrations in the spiked surine samples are depicted by dashed lines. The ZAR-assay based serotonin concentrations were determined by comparison of the emission quenching ratio to that of an independently obtained calibration curve. The deviations of the hereby obtained serotonin concentrations are indicated by the shaded boxes. The excitation wavelength $\lambda_{ex}$ = 395 nm and the emission wavelength $\lambda_{em}$ = 426 nm were used.

Urine samples (spot urine) were collected from healthy voluntary donors spontaneously during the day (morning urine was not used) and used without any pre-treatment steps except for dilution. Samples were stored in aliquots at –20°C. For measurements, samples were defrosted and stored at +4°C and used within 3 - 4 days. Before analysis, samples were incubated at room temperature for 30 minutes. Dilutions were done with water or assay buffer. Eight individual urine samples and spiked samples thereof (30 µM serotonin), respectively, were mixed with **ZAR1** (320 µg $mL^{-1}$, 2.3 wt% dye loading) dispersed in 50 mM HEPES buffer, pH 7.2, and the emission intensity was similarly to the surine assay monitored before and after adding the neurotransmitter solution ($\lambda_{ex}$ = 395 nm; $\lambda_{em}$ = 426 nm). To probe the quenching maximum, 150 µM serotonin were added to each sample and the emission was monitored. The experiments were carried out blinded. Importantly, the analysis of the spiked samples does not require knowledge about the emission response of the corresponding non-spiked urine samples.

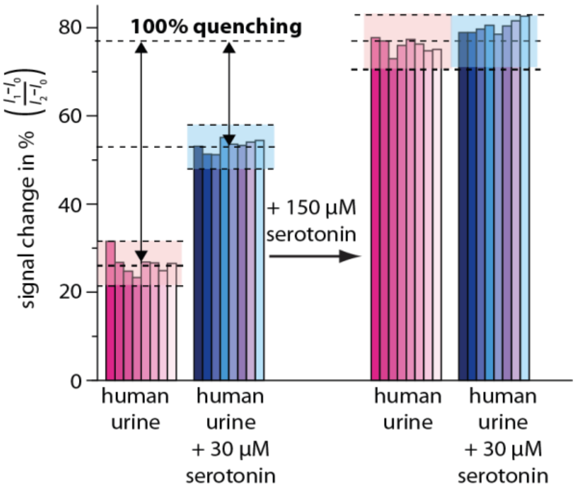


**Fig. S21.** Bar graph of the percentage emission quenching of **ZAR1** (320 µg mL$^{-1}$; 2.3 wt% dye loading) in 50 mM HEPES buffer, pH 7.2, after the addition of urine or spiked urine samples. A **ZAR1**-based assay can distinguish normal from high neurotransmitter levels in human urine samples (n = 8 individual probes). The subsequent addition of 150 µM serotonin was carried out to ensure 100% ZAR-emission quenching, providing a measure for the auto-fluorescence of the urine sample in the presence of the fully emission-quenched ZAR nano-particles. The excitation wavelength $\lambda_{ex}$ = 395 nm and the emission wavelength $\lambda_{em}$ = 426 nm were used.

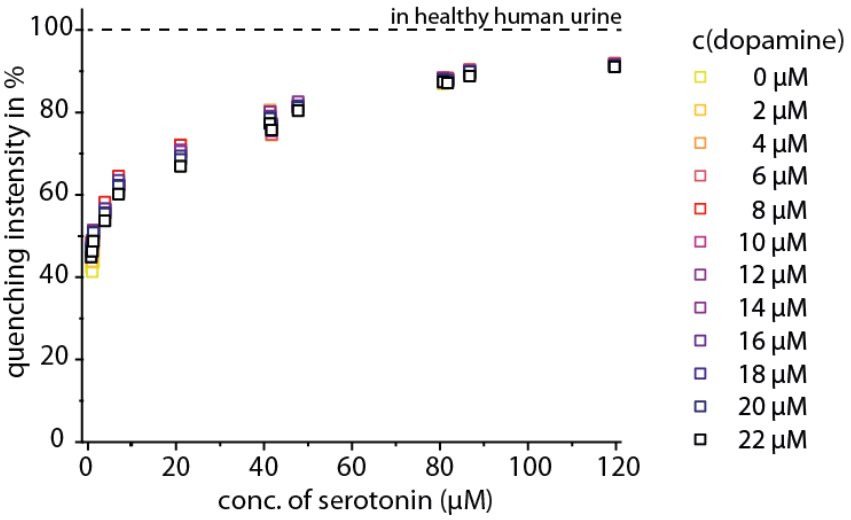


**Fig. S22.** Experimental results for serotonin level determination in serotonin-spiked spot urine samples from healthy volunteers. To simulate the potentially disturbing influence of a simultaneously present but from matrix-to-matrix differing dopamine concentration, these urine samples were also spiked with dopamine (concentration range 0 - 22 µM - equal of 10 times elevated dopamine levels;[31] n = 8 each). Independent of the dopamine concentration, all different serotonin levels are clearly identifiable from each other, rising confidence that serotonin determination in human urine is generally feasible. After a first readout with **ZAR1** (250 µg mL$^{-1}$; 2.3 wt% dye loading) in 25 mM HEPES ($I_0$), human spot urine was added, and the signal detected ($I_1$). The excitation wavelength $\lambda_{ex}$ = 395 nm and the emission wavelength $\lambda_{em}$ = 426 nm were used.

## 9. Thermodynamic characterization of the ZAR-analyte binding process

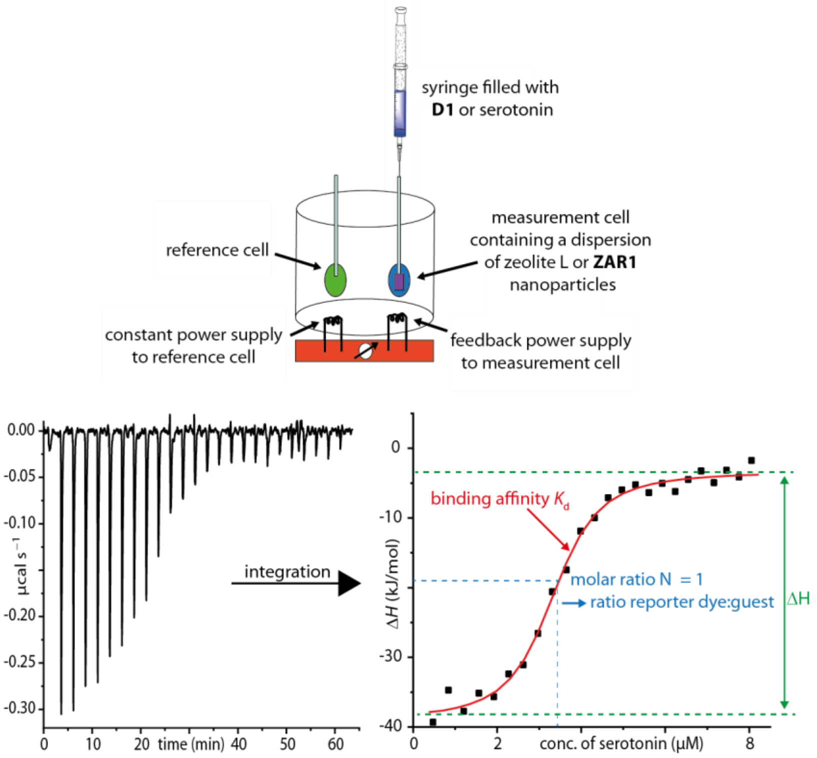


**Fig. S23.** Operation principle of ITC that measures the heat release or heat uptake by the measurement cell containing a solution of a receptor (here an aqueous dispersion of zeolite $L_{3.0}$ nanoparticles or **ZAR1** nanoparticles) upon titration with a solution of a binder (here reporter dye **D1** or serotonin) dissolved in water. The raw binding heats determined by ITC are integrated and corrected for the heat of dilution produced by titrating a **D1** or serotonin solution into water.

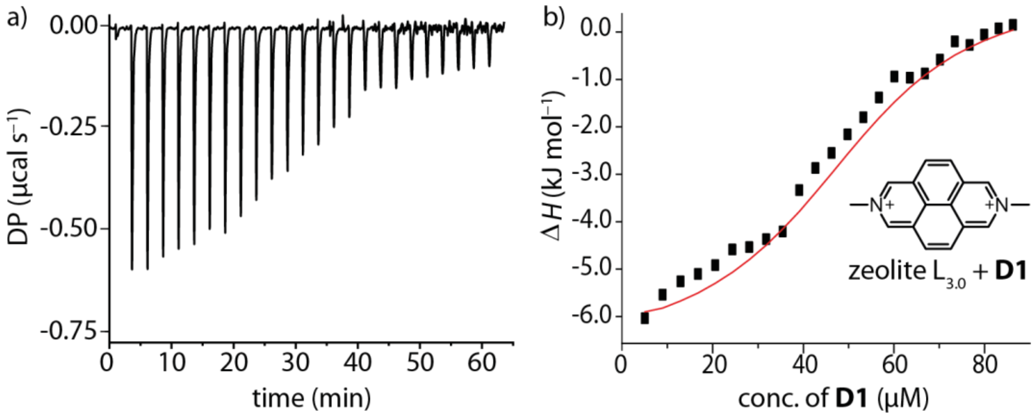


**Fig. S24.** Representative ITC data for the interaction of zeolite $L_{3.0}$ and **D1**. a) Raw ITC data for the titration of **D1** (c = 0 - 85 µM) into a zeolite $L_{3.0}$ dispersion (250 µg $mL^{-1}$). b) Plot for reaction enthalpy vs. concentration of **D1**. The presented data was corrected by the averaged dilution heat determined by dye titration into water and an additional offset was fitted because of the mismatching ionic strength in the host solution compared to the dye solution and

control cell due to bound ions in the zeolite channels. Determined values were: $K_d$ = 0.04 µM, $\Delta H$ = –6.7 kJ mol$^{-1}$; $\Delta G$ = –29.5 kJ mol$^{-1}$, and –T$\Delta S$ = –22.8 kJ mol$^{-1}$.

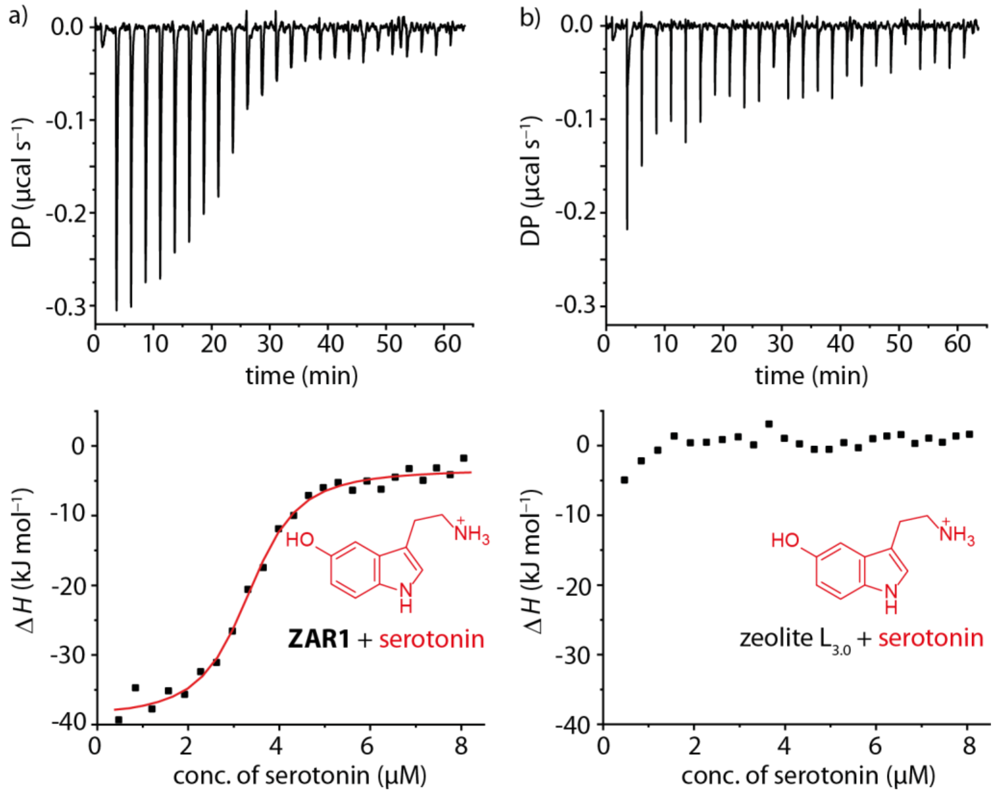


**Fig. S25.** (a) ITC thermogram for the titration of serotonin to **ZAR1** nanoparticles (250 µg mL$^{-1}$; 2.3 wt% dye loading), displaying a strongly exothermic binding signature with a clear 1:1 binding stoichiometry of serotonin to reporter dye **D1**. Serotonin binding to unfilled zeolite cavities of **ZAR1** did not occur to a notable extend. Determined values were: $K_d$ = 0.4 µM, $\Delta H$ = –39.0 kJ mol$^{-1}$, $\Delta G$ = –38.0 kJ mol$^{-1}$, and –T$\Delta S$ = –1.0 kJ mol$^{-1}$. (b) ITC thermogram for the titration of serotonin to zeolite $L_{3.0}$ nanoparticles. The monocationic serotonin binds only weakly ($K_d$ > 1000 µM) to the unfilled zeolite $L_{3.0}$ channels.

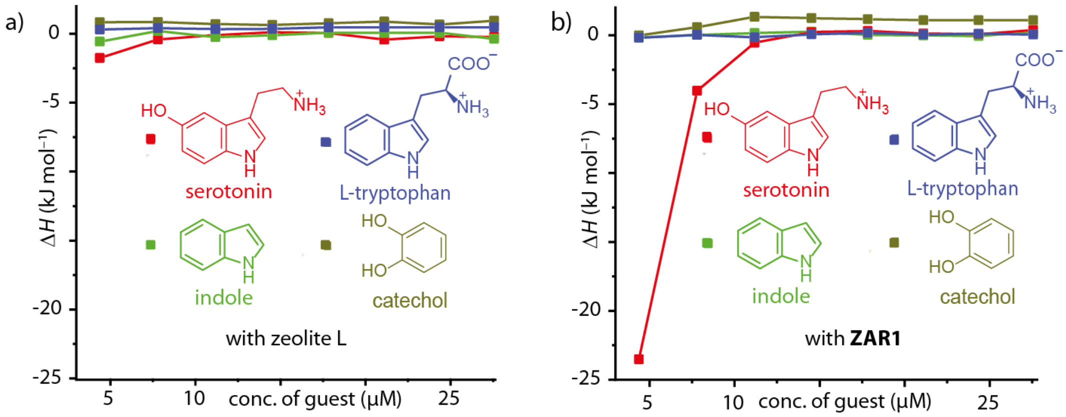


**Fig. S26.** Integrated ITC thermograms for the titration of serotonin (red), L-tryptophan (blue), indole (green) and catechol (olive) to (a) **ZAR1** (250 µg mL$^{-1}$; 2.3 wt% dye loading) and (b) zeolite $L_{3.0}$ (250 µg mL$^{-1}$). Data points are connected by lines to guide the eye. Only serotonin but not its structural analogues showed a sigmoidal binding curve indicative for a strong

binding affinity and a favourable enthalpic binding signature towards **ZAR1**. For zeolite $L_{3.0}$, all guests showed only very weak binding.

## 10. Monitoring of label-free enzymatic reactions in real time

All experiments were conducted under standard assay conditions following the decarboxylase procedures from CREATIVE BIOMART. Specifically, 50 mM HEPES, pH 7.2, and 100 mM sodium chloride were used as assay buffer and pyridoxal 5'-phosphate hydrate (PRP) was used as enzyme cofactor. All stock solutions were prepared in deionized water except for **ZAR2** (final concentration 550 µg $mL^{-1}$; 0.23 wt% dye loading), which was dispersed in 50 mM HEPES buffer, pH 7.2. The enzyme stock concentration was 0.5 mg $mL^{-1}$ (0.365 units $mL^{-1}$). After combining assay buffer, **ZAR2** in HEPES, substrate and PRP, the mixtures were equilibrated for 30 min at 37°C to ensure emission signal stabilization. Then, the enzyme was added to the reaction mixture and the emission was monitored ($\lambda_{ex}$ = 300 nm, $\lambda_{em}$ = 519 nm). If not stated otherwise, final concentrations in the mixture were 45 mM HEPES, pH 7.2, 85 mM sodium chloride, 85 µM PRP, which is in accordance with the underlying decarboxylase assay. For the enzymatic reaction monitoring in neurobasal™ medium, **ZAR2** was directly dispersed in the medium. The enzyme used was native *streptococcus faecalis* tyrosine decarboxylase (TDC) with an activity of 0.73 units $mg^{-1}$, whereas one unit yields 1 µmol of $CO_2$ per minute from L-tyrosine at 37°C, pH 5.5.

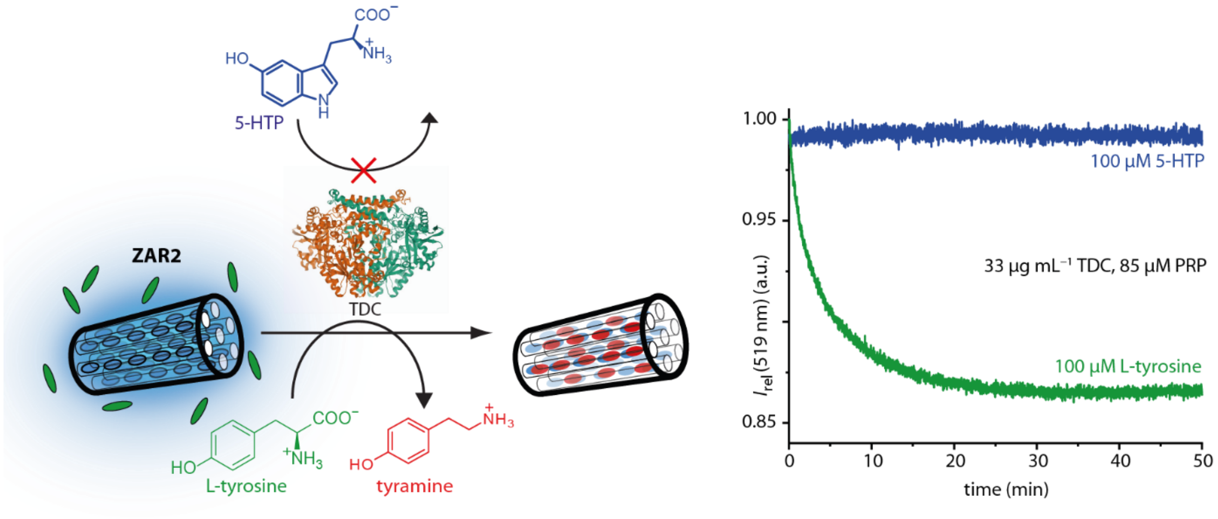


**Fig. S27.** Schematic depiction of the tyrosine decarboxylase (TDC) enzyme-catalyzed decarboxylation of L-tyrosine (Tyr, green), producing the strongly ZAR-binding product tyramine (red). This chemical transformation can be followed in real time by monitoring of the emission intensity response of ZARs that are added to the assay medium. TDC from native *streptococcus faecalis* was reported to specifically decarboxylate Tyr but not 5-HTP (whose decarboxylation product is serotonin). Emission-based reaction monitoring in real time of TDC-catalysed decarboxylation reactions in 45 mM HEPES, pH 7.2, 85 mM sodium chloride with 85 µM pyridoxal 5'-phosphate (PRP) as enzyme cofactor by using **ZAR2** (550 µg mL$^{-1}$; 0.23 wt% dye loading) monitored at $\lambda_{em}$ = 519 nm ($\lambda_{ex}$ = 300 nm). Indeed, TDC does only catalyse the decarboxylation of Tyr but not that of 5-HTP.

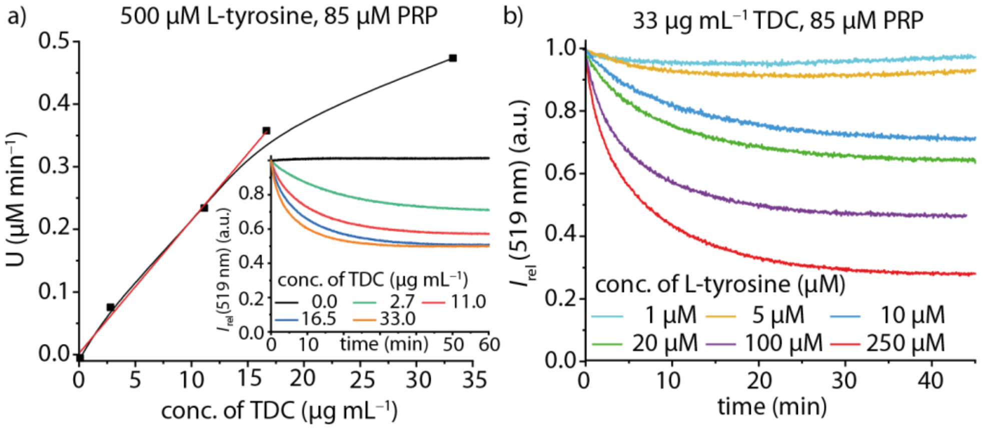


**Fig. S28.** Real-time monitoring of L-tyrosine (c = 500 µM) decarboxylation in the presence of different concentrations of tyrosine decarboxylase (TDC) enzyme, maintaining pyridoxal 5'-phosphate as cofactor (PRP, 85 µM) in excess. The changes in the emission intensity of **ZAR2** (550 µg mL$^{-1}$; 2.3 wt% dye loading) were monitored at $\lambda_{em}$ = 519 nm ($\lambda_{ex}$ = 300 nm). The experiments were carried out under standard assay conditions in 45 mM HEPES buffer, pH 7.2, to which were added 85 mM sodium chloride at 37°C. Initial rates (U) were obtained by linear fitting of the initial signal response regime. Additionally, real-time monitoring of tyrosine decarboxylation catalysed by TDC (33 µg mL$^{-1}$) at six different substrate concentrations, maintaining pyridoxal-5'-phosphate as cofactor (PRP, 85 µM) in excess.

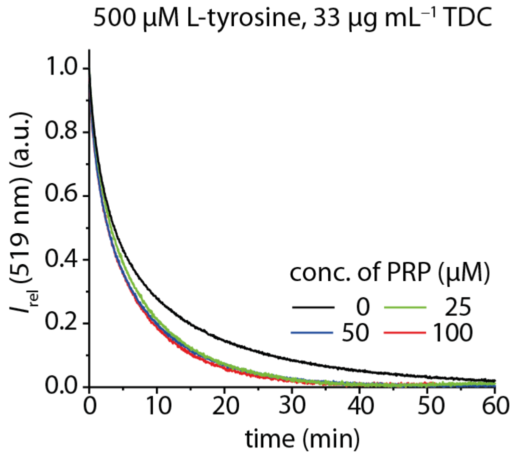


**Fig. S29.** Investigation of the concentration influence of cofactor PRP on the enzyme kinetics of TDC (33 µg mL$^{-1}$). Clearly, 25 µM suffice to ensure maximum enzymatic activity of 33 µg mL$^{-1}$ TDC. The experiments were carried out under standard assay conditions in 45 mM HEPES buffer, pH 7.2, to which were added 85 mM sodium chloride at 37°C. The changes in the emission intensity of **ZAR2** (550 µg mL$^{-1}$; 2.3 wt% dye loading) were monitored at $\lambda_{em}$ = 519 nm ($\lambda_{ex}$ = 300 nm).

## 11. Functionality of ZARs in organic solvents

An interesting and potentially useful feature of the ZARs is their full functionality in organic solvents. Thus, analytical protocols that pre-extract the neurotransmitter in an organic solvent, *e.g.*, as its free base, are combinable with our fluorescent neurotransmitter detection strategy. Fundamentally important, a higher binding affinity for the protonated serotonin was observed in aqueous than in ethanolic media (10x stronger for **ZAR1**). Based on a lock-and-key argument, the opposite would have been expected because salt-bridges are stronger in a less polar environment. In addition, the energetic cost of desolvation of protonated serotonin from the bulk solvent is higher in water than in ethanol.[32] Nevertheless, the affinity is substantially higher in the more cohesive solvent water, which is a good indication for the importance of the non-classical hydrophobic effect (*i.e.*, high-energy cavity water molecules) in the zeolite-based receptor system.[33]

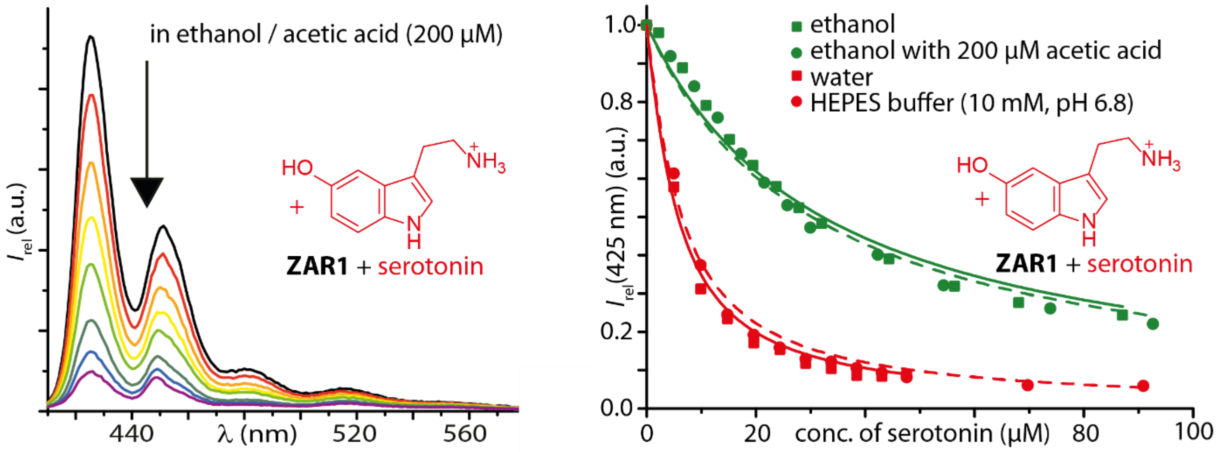


**Fig. S30.** Titration of **ZAR1** (250 μg $mL^{-1}$; 0.23 wt% dye loading) with serotonin in ethanol (in the presence and absence of 0.2 mM acetic acid). The excitation wavelength $\lambda_{ex}$ = 396 nm was used. Fit of the relative emission intensity with a 1:1 binding site model yields a single site dissociation constant $K_d \leq 2$ μM in water and aqueous buffer, and $K_d \sim 25$ μM in ethanolic media. The weaker binding affinity in ethanol than in aqueous media indicates a favourable hydrophobic contribution to binding in water. Conversely, if the energetic contributions of salt bridges and cation-π interactions were dominating, stronger binding affinities in ethanol than in water would have been expected.

## 12. Theoretical methods

The structural and optical properties of **D1** and serotonin were studied using the density functional theory (DFT) method with the hybrid PBE0 functional.[34] For all atoms, the triple-ζ-valence-plus-polarization (def2-TZVP) atomic orbital (AO) basis sets were used.[35] The Grimme D3 dispersion correction with Becke-Johnson damping was included for all systems studied.[36,37] Both molecules, together with their complexes, were fully optimized using minimization techniques and absorption spectra were simulated with COSMO[38] model for water. All structures were characterized as local minima through harmonic vibrational analysis.

The "full" zeolite model was composed of two zeolite $L_{3.0}$ pores, **D1** and serotonin molecules surrounded with 24 $H_2O$ molecules, and was optimized by applying the resolution of identity (RI)-DFT procedure[39] together with multipole accelerated resolution of identity-J ("marij") scheme. The non-hybrid Perdew-Burke-Ernzerhof (PBE) functional[40] and def2-SVP AO basis set[41] was used for all atoms to considerably reduce computational complexity. The Grimme

D3 dispersion correction was included. Converged structural configuration of the dye and analyte within the zeolite channel were extracted and used to simulate optical properties during their interaction using time-dependent density functional theory (TD-DFT) as described further.

To simulate the one-photon absorption properties of interacting **D1** and serotonin, we calculated 30 lowest-lying singlet transitions and applied Lorentzian broadening with full-width at half maximum (FWHM) of 15 nm. All calculations were performed within TURBOMOLE 7.3 code. Vibrationally-resolved electronic absorption spectra for **D1** were simulated using Gaussian16 software with Franck-Condon-Herzberg-Teller method[42] and the same PBE0 functional, def2-TZVP basis set, Grimme D3 correction with Becke-Johnson damping and PCM model for water.

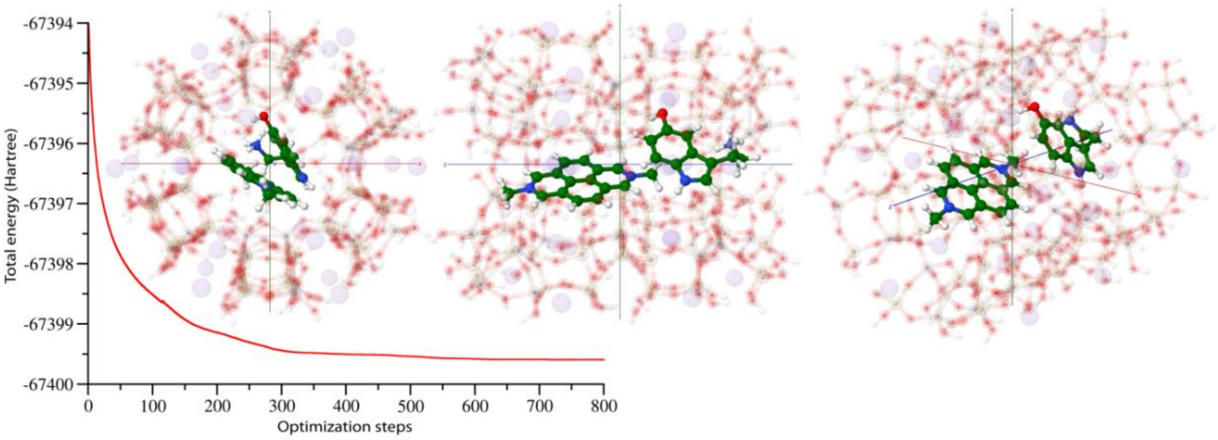


**Fig. S31.** DFT optimized zeolite $L_{3.0}$ model with 27 substituted Si/Al atoms including 24 $K^+$ ions, 24 $H_2O$ molecules, **D1** and serotonin molecules interacting within the zeolite channel (view along z- and x-axis and perspective view).

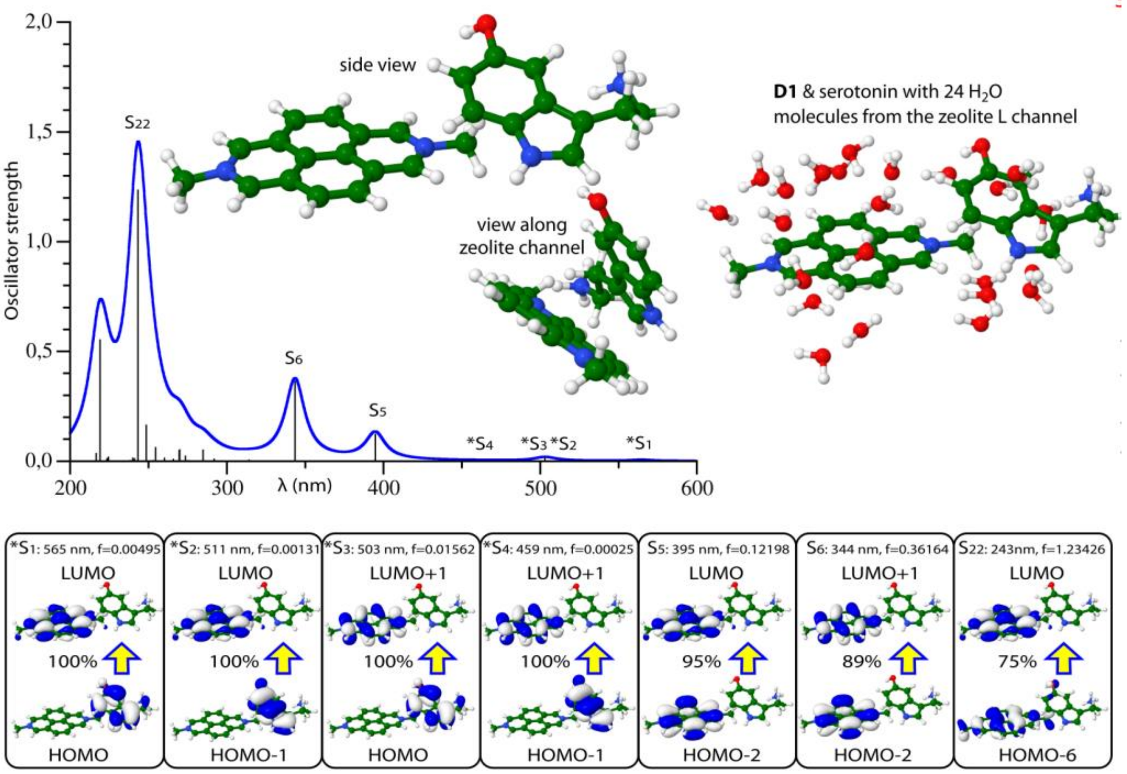


**Fig. S32.** TD-DFT simulated absorption spectrum of **D1** and serotonin based on calculated discrete vertical transitions (black vertical sticks), with the same dye and analyte orientation as in the zeolite channel with an implicit COSMO water environment. Analysis of the leading contributions to the main bands shows additional low-lying charge transfer transitions $S_1$-$S_4$ responsible for quenching mechanism marked with an asterisk (*).

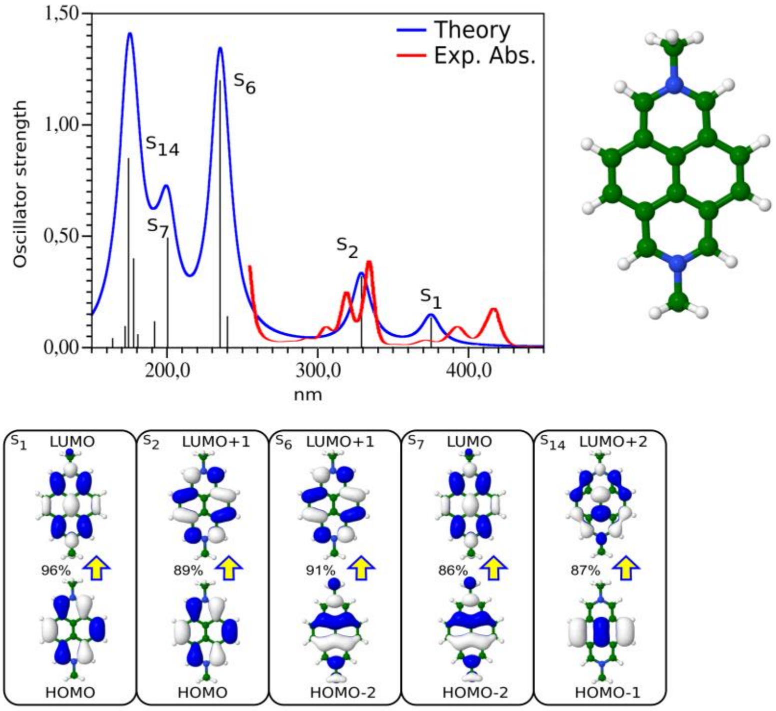


**Fig. S33.** TD-DFT simulated absorption spectrum of **D1** in the implicit water COSMO model. Analysis of the contributing orbitals of the leading transitions (black sticks) shows which

electrons are contributing to the absorption properties of **D1**. Cut-off for MOs is 0.03, minus and plus are labelled by blue and white colors, respectively (the blue full line corresponds to Lorentzian broadening with full width at half maximum (FWHM) of 15 nm, the black vertical lines correspond to values of oscillator strength).

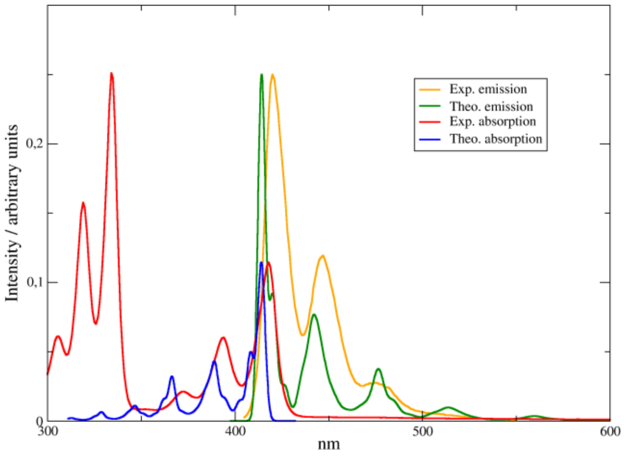


**Fig. S34.** TD-DFT simulated absorption and emission spectrum of **D1** in the implicit water COSMO model as comparison of the simulated vibrationally resolved spectra with experimental measurements for **D1**.

<u>Charge transfer analysis during electron excitation based on the electron density difference</u>

Analysis of charge transfer (CT) properties during electron transfer from density functional theory calculations were based on a 3D generalized approach described in *J. Chem. Theory Comput.*, 7, 2498 (2011) which is implemented in the MultiWfn 3.6dev software. Theoretical details are described in chapters 3.21.3 and 4.18.3 of the program's manual. Quantification of charge transfer between the molecules first required a calculation of electron density difference between each excited state $\rho_{EX}(r)$ and ground state $\rho_{GS}(r)$:

$$\Delta\rho(r) = \rho_{EX}(r) - \rho_{GS}(r) \quad \text{Eq. S9}$$

For ground state $S_0$ and each singlet excited state $S_1$-$S_5$ we saved converged density in Gaussian16 program of the ground state structure meaning that we performed only vertical excitation analysis. After performing subtraction of the densities, electron density difference $\boldsymbol{\Delta\rho(r)}$ is partitioned into positive $\rho_+$ and negative $\rho_-$ parts using MultiWfn 3.6dev program. The integral of each partitioned electron density difference gave the amount of transferred

charge $q_{CT}$ which, in principle, can have a value different than the full number due to the fact that excitation of the one electron leads to the reorganization of the other electrons in the system which is also accounted within each state's density. Barycentres of positive and negative parts of electron density difference were also determined and their respective distance represents the CT length.

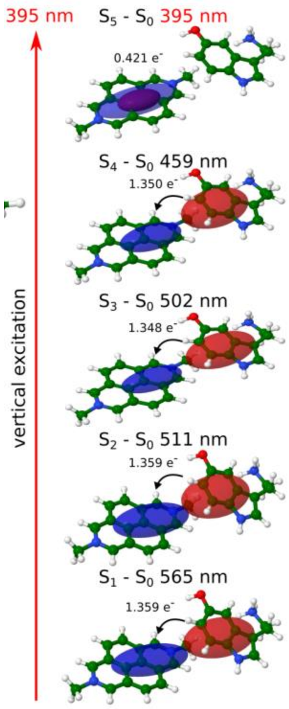


**Fig. S35.** Charge transfer analysis based on the electron density difference between each of the first five excited states $S_1$ - $S_5$ and the ground state $S_0$ showing clearly that additional low-lying transitions can be characterized as a charge transfer transitions.

# 13. Further scope of ZARs

## 13.1. Detection of non-quenching neurotransmitters, *e.g.*, histamine

An important feature of the confinement of the dye into the zeolite is the possibility to form dye aggregates in the channels. Upon aggregation some dyes display an exciplex emission at low energy that is well separated from the monomeric fluorescence. This behaviors is enhanced inside the zeolite nanopores. Disaggregation of the dyes can be used for the detection of analytes that cannot quench the emission of the dyes. Addition of the non-

quenching neurotransmitter histamine causes a dye-deaggregation of **D3** that is signaled by a shift in the intensity ratio at different wavelengths, respectively.

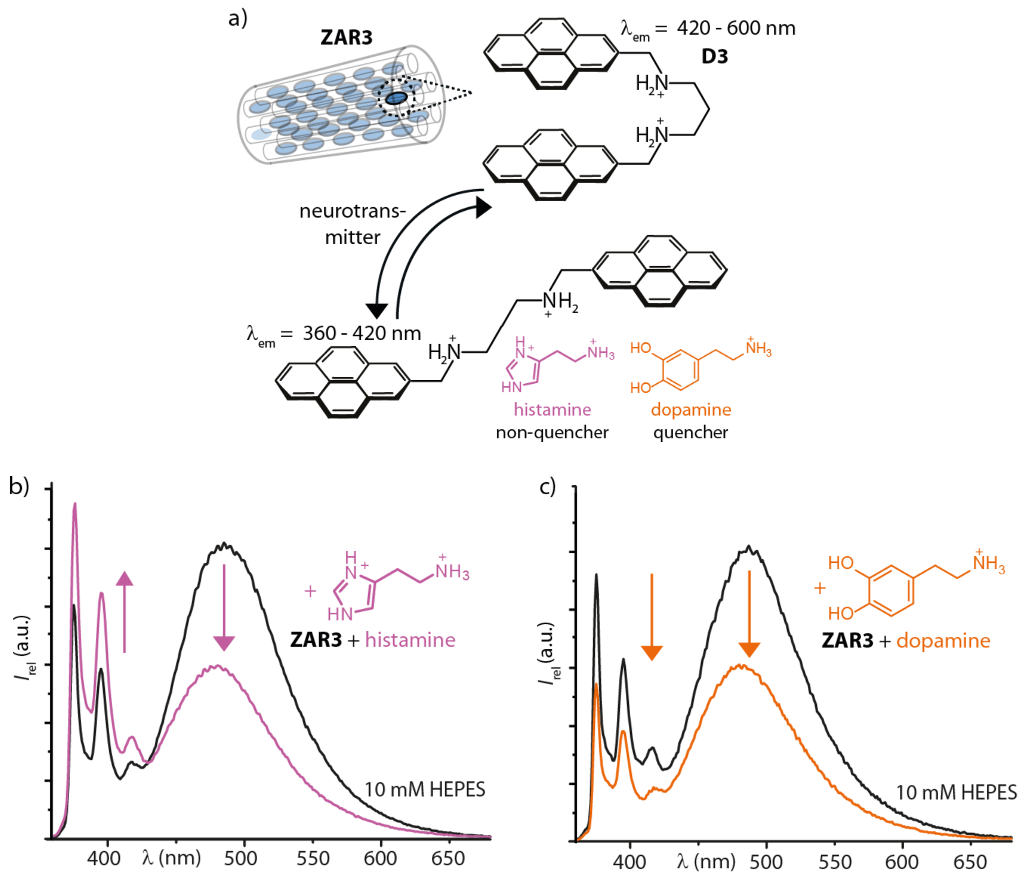


**Fig. S36.** (a) Schematic representation of the signal transduction mechanism that depicts the proposed unfolding of reporter dye **D3** upon capture of the neurotransmitters by **ZAR3**. (b) Ratiometric emission spectra response of **ZAR3** (250 µg mL$^{-1}$; 0.23 wt% dye loading; **D3** = $N^1$,$N^3$-bis(pyren-1-ylmethyl)propane-1,3-diaminium) to the presence of the electron-poor histamine (c = 120 µM) as non-quencher and the electron-rich dopamine (c = 85 µM) as PET-quencher in 10 mM HEPES buffer ($\lambda_{ex}$ = 445 nm).

### 13.2. Fluorescence turn-on assays

The large dye **D2** shows signs for self-aggregation in the larger-cavity of zeolite $Y_{2.55}$, which allows for the detection of histamine binding to **ZARY$_{2.55}$2**. The addition of the non-quenching neurotransmitter histamine causes a dye-deaggregation that is signaled by a clear increase in the emission intensity.

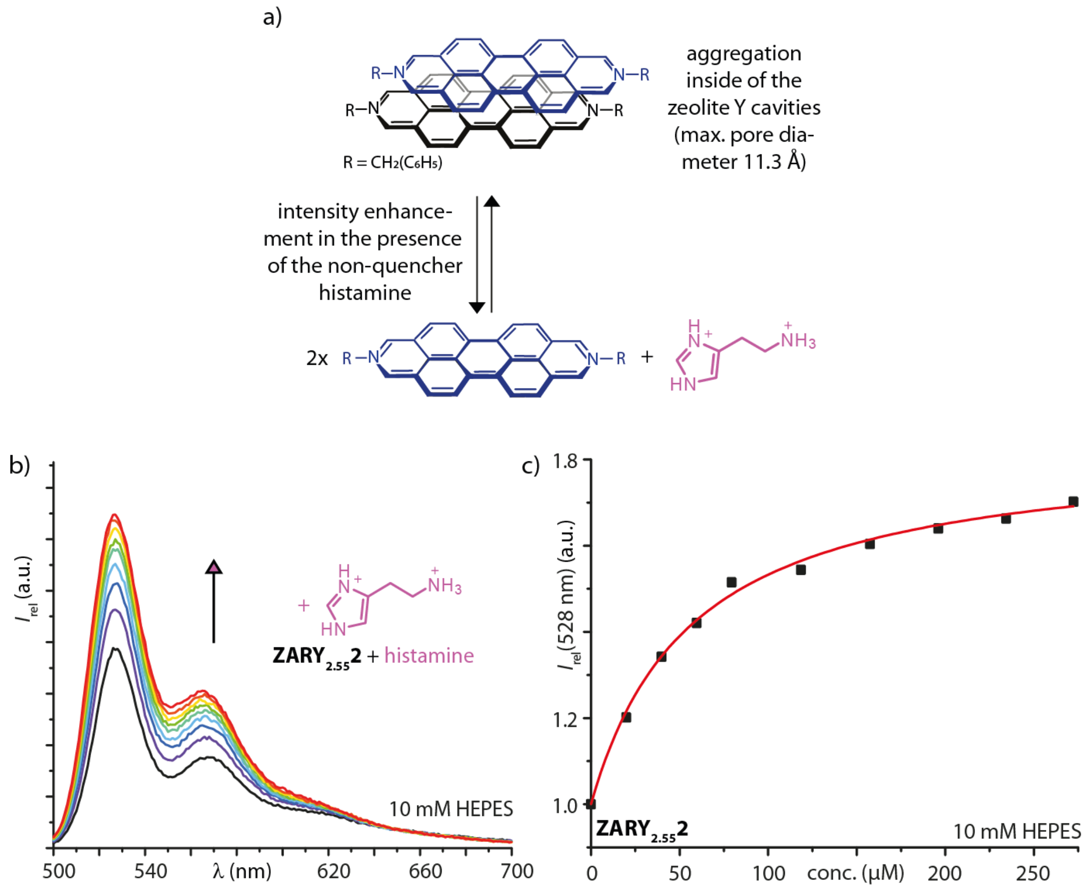


**Fig. S37.** Emission-based titration of **ZARY$_{2.55}$2** (250 µg mL$^{-1}$; 0.23 wt% dye), composed of zeolite Y with a Si-to-Al-ratio of 2.55 that was loaded with reporter dye **D2**, with the electron-poor neurotransmitter histamine in 10 mM HEPES, pH 7.3, and the corresponding emission-based binding curve ($\lambda_{ex}$ = 475 nm). Fitting of the binding isotherm yielded an affinity of $K_d$ = 56 µM.

### 13.3. Detection of neutral and zwitterionic analytes

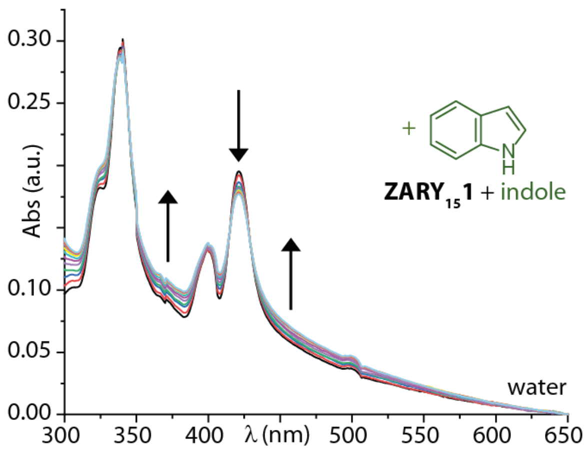


**Fig. S38.** Absorbance spectra for the titration of **ZARY$_{15}$1** (250 µg mL$^{-1}$; 2.3 wt% dye loading), composed of zeolite Y with a Si-to-Al-ratio of 15 that was loaded with reporter dye **D1**, with the non-charged analyte indole in water.

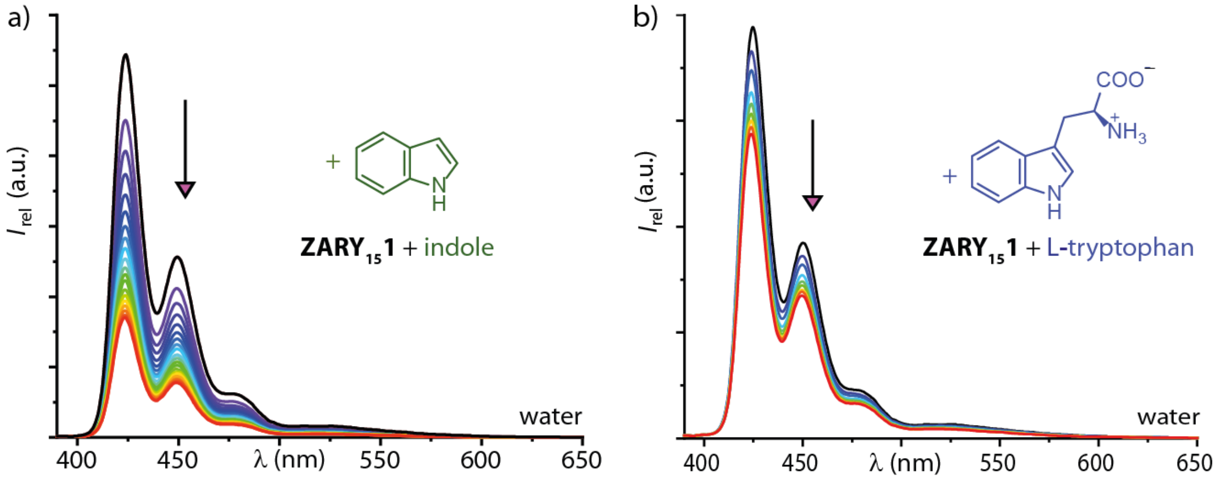


**Fig. S39.** Emission spectra of the titration of **ZARY$_{15}$1** (250 µg mL$^{-1}$; 2.3 wt% dye loading), composed of zeolite Y with a Si-to-Al-ratio of 15 that was loaded with reporter dye **D1**, with (a) the non-charged indole and (b) the zwitterionic L-tryptophan in water ($\lambda_{ex}$ = 371 nm).

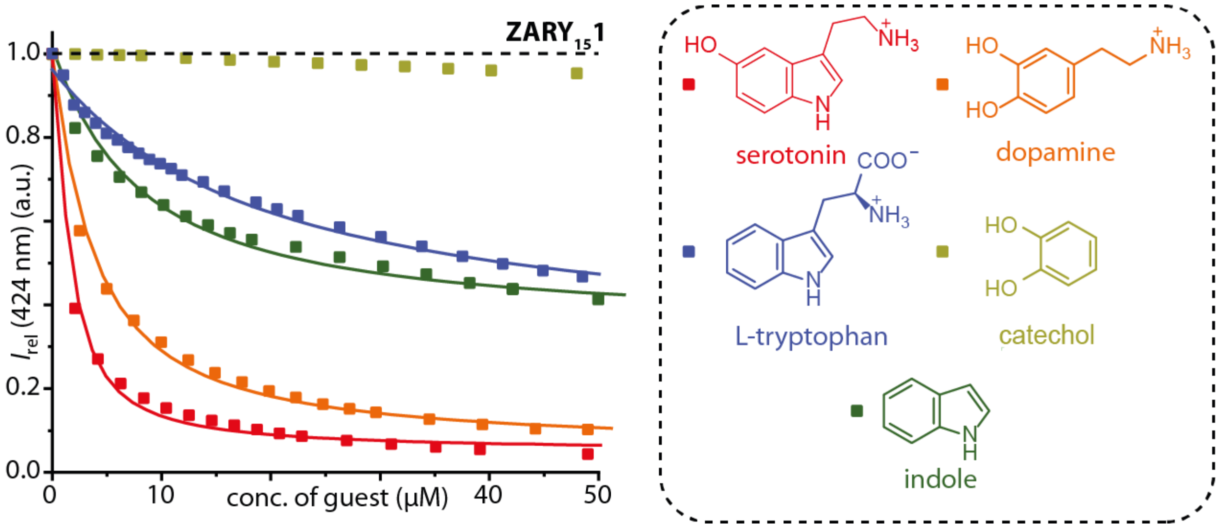


**Fig. S40.** Emission-based binding curves for several neurotransmitters and structural related molecules to **ZARY$_{15}$1** (250 µg mL$^{-1}$; 0.23 wt% dye loading) in water ($\lambda_{ex}$ = 371 nm) fitted with a 1:1 binding model.

## 14. Synthesis

*Synthesis of N$^1$,N$^3$-bis(pyren-1-ylmethyl)propane-1,3-diaminium diacetate* (**D3**):

1-Pyrene-carboxaldehyde (250 mg, 1.10 µmol, 2.2 eq) was dissolved in MeCN (10 mL) and 1,3-diaminopropane (37.0 mg, 500 nmol, 1.0 eq) was added. The solution was cooled in an ice bath. Sodium cyanoborohydride (150 mg, 2.50 µmol, 5.0 eq) was slowly added and the reaction mixture was stirred for 1 h by allowing it to warm up to room temperature. Then, two drops of acetic acid (glacial) were added, and then another two drops after 2 h total reaction time. The reaction mixture was stirred overnight, and the reaction progress was followed by TLC. Diethyl ether (200 mL) was added, and the organic phase was washed with aqueous sat. sodium carbonate (2x 100 mL) and with water (100 mL). The organic phase was dried over sodium sulfate and the solvent was evaporated under reduced pressure. The residue was taken up in a minimal amount of chloroform and reprecipitated upon addition of a few drops of acetic acid. The title compound was obtained by suction filtration, followed by washing with diethyl ether and drying under reduced pressure to yield an off-white solid 70% (217 mg, 349 nmol). $^{1}$H NMR (400 MHz, DMSO-$d_6$, δ): 8.41 (d, 2H), 8.27 - 8.01 (m, 20H), 4.36 (s, 4H), 2.77 (t, 4H), 1.74 (s, 6H, -$CH_3$ of acetate) ppm; $^{13}$C NMR (101 MHz, DMSO-$d_6$, δ):

174.17 (-COO of acetate), 135.4 (Cq), 131.3 (Cq), 130.8 (CH), 130.2 (Cq), 129.0 (CH), 127.9 (CH), 127.5 (Cq), 127.4 (CH), 127.2 (CH), 126.5 (CH), 125.4 (CH), 125.3 (CH), 125.0 (CH), 124.5 (CH), 124.2 (CH), 51.6 ($CH_2$), 48.45 ($CH_2$), 23.95 (-$CH_3$ of acetate) ppm; ESI-MS *m/z*: $[M\text{-}H]^+$ calcd for $C_{37}H_{31}N_2^+$ 503.25; found 503.25.

## 15. References


(1) H. E. Gottlieb, V. Kotlyar, A. Nudelman, *J. Org. Chem.* **1997**, *62* (21), 7512.
(2) G. R. Fulmer, A. J. Miller, N. H. Sherden, H. E. Gottlieb, A. Nudelman, B. M. Stoltz, J. E. Bercaw, K. I. Goldberg, *Organometallics* **2010**, *29* (9), 2176.
(3) J. Knabbe, J. P. Nassal, M. Verhage, T. Kuner, *J. Physiol.* **2018**, *596* (16), 3759.
(4) S. Megelski, G. Calzaferri, *Adv. Funct. Mater.* **2001**, *11* (4), 277.
(5) C. Baerlocher, L. B. McCusker, D. H. Olson, W. M. Meier *Atlas of zeolite framework types*; Published on behalf of the Structure Commission of the International Zeolite Association by Elsevier: Amsterdam, **2007**.
(6) C. Baerlocher, L. B. McCusker, “Database of Zeolite Structures”, http://www.iza-structure.org/databases/, 2017, accessed: 07, **2021**.
(7) E. Fois, G. Tabacchi, G. Calzaferri, *J. Phys. Chem. C* **2010**, *114* (23), 10572.
(8) B. Boddenberg, G. U. Rakhmatkariev, S. Hufnagel, Z. Salimov, *Phys. Chem. Chem. Phys.* **2002**, *4* (17), 4172.
(9) H. S. Sherry, *J. Phys. Chem.* **1966**, *70* (4), 1158.
(10) R. P. Townsend, E. N. Coker, in *Stud. Surf. Sci. Catal.*, Vol. 137 (Eds. H. van Bekkum, E. M. Flanigen, P. A. Jacobs, J. C. Jansen), Elsevier, **2001**.
(11) M. M. Lozinska, D. N. Miller, S. Brandani, P. A. Wright, *J. Mater. Chem. A* **2020**, *8* (6), 3280.
(12) M. Herm, O. Molt, T. Schrader, *Angew. Chem. Int. Ed.* **2001**, *40* (17), 3148.
(13) F. Biedermann, W. M. Nau, H.-J. Schneider, *Angew. Chem. Int. Ed.* **2014**, *53* (42), 11158.
(14) G. Campiani, E. Morelli, S. Gemma, V. Nacci, S. Butini, M. Hamon, E. Novellino, G. Greco, A. Cagnotto, M. Goegan, L. Cervo, F. Dalla Valle, C. Fracasso, S. Caccia, T. Mennini, *J. Med. Chem.* **1999**, *42* (21), 4362.
(15) F. J. Bolaños, L. E. Schechter, M. C. Miquel, M. B. Emerit, J. F. Rumigny, M. Hamon, H. Gozlan, *Biochem. Pharmacol.* **1990**, *40* (7), 1541.
(16) L. Rivail, M. Giner, M. Gastineau, M. Berthouze, J.-L. Soulier, R. Fischmeister, F. Lezoualc'h, B. Maigret, S. Sicsic, I. Berque-Bestel, *Br. J. Pharmacol.* **2004**, *143* (3), 361.
(17) R. K. Sunahara, H.-C. Guan, B. F. O'Dowd, P. Seeman, L. G. Laurier, G. Ng, S. R. George, J. Torchia, H. H. M. Van Tol, H. B. Niznik, *Nature* **1991**, *350* (6319), 614.
(18) K. Nishikori, O. Noshiro, K. Sano, H. Maeno, *J. Biol. Chem.* **1980**, *255* (22), 10909.
(19) H. Maeno, *Mol. Cell. Biochem.* **1982**, *43* (2), 65.
(20) S. W. Robinson, K. R. Jarvie, M. G. Caron, *Mol. Pharmacol.* **1994**, *46* (2), 352.
(21) F. Lanau, M.-T. Zenner, O. Civelli, D. S. Hartman, *J. Neurochem.* **1997**, *68* (2), 804.

(22) G. Calzaferri, S. Huber, H. Maas, C. Minkowski, *Angew. Chem. Int. Ed.* **2003**, *42* (32), 3732.
(23) A. R. Bernardo, J. F. Stoddart, A. E. Kaifer, *J. Am. Chem. Soc.* **1992**, *114* (26), 10624.
(24) O. Molt, D. Rübeling, G. Schäfer, T. Schrader, *Chemistry* **2004**, *10* (17), 4225.
(25) C. Givelet, B. Bibal, *Org. Biomol. Chem.* **2011**, *9* (21), 7457.
(26) T. Nyrönen, M. Pihlavisto, J. M. Peltonen, A.-M. Hoffrén, M. Varis, T. Salminen, S. Wurster, A. Marjamäki, L. Kanerva, E. Katainen, L. Laaksonen, J.-M. Savola, M. Scheinin, M. S. Johnson, *Mol. Pharmacol.* **2001**, *59* (5), 1343.
(27) C. A. Cornil, G. F. Ball, *J. Comp. Neurol.* **2008**, *511* (5), 610.
(28) S. Kasera, L. O. Herrmann, J. d. Barrio, J. J. Baumberg, O. A. Scherman, *Sci. Rep.* **2014**, *4* (1), 6785.
(29) R. H. Bisby, S. W. Botchway, S. Dad, A. W. Parker, *Photochem. Photobiol. Sci.* **2006**, *5* (1), 122.
(30) F. Biedermann, D. Hathazi, W. M. Nau, *Chem. Commun.* **2015**, *51* (24), 4977.
(31) S. Bouatra, F. Aziat, R. Mandal, A. C. Guo, M. R. Wilson, C. Knox, T. C. Bjorndahl, R. Krishnamurthy, F. Saleem, P. Liu, Z. T. Dame, J. Poelzer, J. Huynh, F. S. Yallou, N. Psychogios, E. Dong, R. Bogumil, C. Roehring, D. S. Wishart, *PLOS ONE* **2013**, *8* (9), e73076.
(32) The better solvation of protonated serotonin in aqueous buffer than in ethanol is evident by the approx. 3x higher solubilty of serotonin in water than in ethanol, see for instance Santa Cruz Biotechnology product sheet for Serotonin hydrochloride (CAS 153-98-0).
(33) D. B. Smithrud, F. Diederich, *J. Am. Chem. Soc.* **1990**, *112* (1), 339.
(34) J. P. Perdew, M. Ernzerhof, K. Burke, *J. Chem. Phys.* **1996**, *105* (22), 9982.
(35) A. Schäfer, C. Huber, R. Ahlrichs, *J. Chem. Phys.* **1994**, *100* (8), 5829.
(36) S. Grimme, J. Antony, S. Ehrlich, H. Krieg, *J. Chem. Phys.* **2010**, *132* (15), 154104.
(37) S. Grimme, S. Ehrlich, L. Goerigk, *J. Comput. Chem.* **2011**, *32* (7), 1456.
(38) A. Klamt, G. Schüürmann, *J. Chem. Soc., Perkin Trans. 2* **1993**, (5), 799.
(39) K. Eichkorn, O. Treutler, H. Öhm, M. Häser, R. Ahlrichs, *Chem. Phys. Lett.* **1995**, *240* (4), 283.
(40) J. P. Perdew, K. Burke, M. Ernzerhof, *Phys. Rev. Lett.* **1996**, *77* (18), 3865.
(41) F. Weigend, R. Ahlrichs, *Phys. Chem. Chem. Phys.* **2005**, *7* (18), 3297.
(42) F. Santoro, A. Lami, R. Improta, J. Bloino, V. Barone, *J. Chem. Phys.* **2008**, *128* (22), 224311.
